# Effect of microstructure on hydrogen permeation and trapping in natural gas pipeline steels

**DOI:** 10.1038/s41529-025-00615-5

**Published:** 2025-06-14

**Authors:** Aminul Islam, Qidong Li, Emma Storimans, Kay Ton, Tahrim Alam, Zoheir N. Farhat

**Affiliations:** 1 National Research Council Canada, Clean Energy Innovation Research Centre, Vancouver, BC Canada; 2 Dalhousie University, Department of Mechanical Engineering, 1360 Barrington Street, Halifax, NS Canada; 3 Enbridge Gas Inc., Low Carbon Transition Engineering, Ottawa, ON Canada

**Keywords:** Engineering, Materials science

## Abstract

This study examines hydrogen permeation and trapping in three types of natural gas pipeline steels from different decades in Canada—modern, vintage, and legacy steels. Electrochemical permeation experiments were conducted to measure the diffusion coefficient, subsurface concentration, and trap density of hydrogen. The results were analyzed to evaluate the susceptibility of these steels to hydrogen embrittlement and to understand the effects of hydrogen on their mechanical properties. Vintage steel exhibited 50% higher steady-state permeation current and 97% greater effective diffusivity compared to modern steel, while legacy steel showed intermediate values. Hydrogen diffusion increased with grain size and pearlite content but decreased with dislocation density. Modern steel demonstrated the highest resistance to hydrogen permeation due to its finer grain structure and higher dislocation density. This study provides essential insights into the diffusion behavior and trapping mechanisms of hydrogen in natural gas pipeline steels, enhancing the understanding of material performance under hydrogen exposure.

## Introduction

To reach the goal of net-zero emissions by 2050, the world is transitioning from fossil fuels to renewable energy sources such as wind, hydro, solar, and geothermal^1,2^. However, these sources are not universally available due to geographical and environmental constraints, necessitating the exploration of alternative energy options. Hydrogen, recognized as a clean energy carrier, is considered critical for achieving a climate-neutral and pollution-free economy. There are multiple, technologically mature methods to produce hydrogen, including natural gas reforming, electrolysis of water, and coal gasification^3^. One way to utilize hydrogen is by blending it with natural gas and distributing it through existing natural gas pipelines. Although natural gas has the lowest carbon emissions of all fossil fuels, it still contains a significant amount of carbon. Blending hydrogen with natural gas can create a less carbon-intensive alternative, as the combustion of hydrogen produces only water, making it an exceptionally clean.

Implementation of hydrogen blends into the natural gas network for homes and industrial applications can impact existing and new pipeline networks. Hydrogen can induce an environmentally assisted failure of steel pipelines mostly due to the combined action of hydrogen and residual and applied stress. This phenomenon is known as hydrogen embrittlement (HE) and may lead to catastrophic failures^4–7^. Introduction of hydrogen in natural gas pipeline can cause significant degradation in mechanical properties, notably toughness and ductility^8^. Islam et al.^9^ performed a comprehensive review of material compatibility for hydrogen blending in natural gas. Although the specific mechanism of hydrogen embrittlement can be different depending on the microstructure, applied stress and environmental conditions and multiple mechanisms can act simultaneously for the failure, it is well-established that hydrogen enters the metal through processes such as physisorption, chemisorption, and uptake^10–13^. One of the important factors that need to be understood for the pipeline is the permeation and diffusion behavior. Electrochemical hydrogen permeation, a method pioneered by Devanathan and Stachurski, can be used to investigate this phenomenon^14^.

There are numerous factors that influence hydrogen permeation behavior. These include environmental factors such as pH and applied voltage or current, as well as characteristics of the metal, such as dislocation density, grain size, microstructure, and alloy precipitates. These microstructural features can act as potential trap sites for hydrogen and influence permeation behavior. Hydrogen traps have the capacity to bind with hydrogen and include vacancies, solute atoms, inclusions, precipitation phases, grain boundaries, and various crystallographic phases. Traps are classified as either reversible or irreversible. Reversible traps allow hydrogen to escape before saturation is reached, while irreversible traps mean that the trapped hydrogen cannot escape^15^. The increase in hydrogen concentration due to trapping is a crucial factor in the brittle failure of metals and is essential for the understanding of embrittlement phenomena.

Cold working, external stresses and uneven cooling rates induces dislocations in metals^16^. Jun et al.^17^ investigated hydrogen embrittlement in iron using molecular dynamics simulations. Their findings revealed a transition from ductile to brittle behavior, driven by the suppression of dislocation emission at the crack tip due to hydrogen accumulation. This suppression leads to brittle cleavage failure, followed by slow crack growth as hydrogen clusters at the crack tip, weakening the material’s resistance to fracture. The study by Bulatov and Cai^18^ discusses the direct observation of hydrogen-enhanced dislocation mobility in iron using in situ electron microscopy focusing on how hydrogen assists in the motion of dislocations. The research confirms that hydrogen increases dislocation mobility in BCC metals, which may contribute to embrittlement.

Generally, the diffusion rate of hydrogen decreases because dislocations can act as potential hydrogen traps. However, dislocations are considered weak hydrogen traps because of their low binding energy^19^. In a study by Song et al.^20^, cold working through laser peening (LP) was applied, and its effect on the hydrogen permeation rate was investigated. The results showed that metals subjected to laser peening exhibited increased dislocations and more tortuous grain boundaries, leading to a reduction in hydrogen permeation. Conversely, research by Martin et al.^21^ highlighted that hydrogen is particularly sensitive to the elastic stress field surrounding dislocations. This is because of the hydrogen-dislocation drag, triggered by an external force, which causes the hydrogen to migrate along these dislocations^15,22–24^.

The impact of grain size and grain boundaries on hydrogen diffusion has been extensively studied by various researchers^25–28^. A reduction in grain size increases the grain boundary area per unit volume within the microstructure. It is commonly believed that hydrogen is preferentially trapped at grain boundaries, which can delay its migration. Additionally, an increase in grain boundaries has been associated with a rise in the density of triple junctions, further slowing hydrogen diffusion^29^. For example, based on varying grain sizes, Ichimura et al.^30^ developed a model to calculate the diffusivity of hydrogen in aluminum. Their results showed that smaller grains lead to reduced diffusivity due to extensive hydrogen trapping at grain boundary nodes. Conversely, larger grains result in increased diffusivity as hydrogen diffuse along the grain boundaries, a phenomenon known as short-circuit diffusion^31^. Ramunni et al.^26^ found that, at high temperatures, grain size does not significantly affect hydrogen diffusion in bcc iron. However, at room temperature (290 K) and for nano-sized grains (100 nm), the effective diffusion rate can decrease by up to two orders of magnitude. Although grain boundaries are typically considered hydrogen traps, they can, in some cases, facilitate diffusion, as demonstrated by Oudriss et al.^32^ and Brass et al.^28^ in their studies on polycrystalline nickel. Yazdipour et al.^33^ investigated the effect of grain size on hydrogen diffusion in API X70 steel using 2D modeling and validated their findings by comparing simulation results with experimental measurements of hydrogen permeability in API X70 pipeline steel with varying grain sizes. Their results support the hypothesis that grain boundaries can have opposing effects on the diffusivity of hydrogen atoms.

Steel microstructures significantly affect hydrogen diffusion rates. Various studies have investigated the impact of microstructural features on hydrogen diffusion rates^34–41^. Park et al.^42^ modified the microstructure of API X65 pipeline steel through heat treatment and studied its effect on hydrogen permeability. Their findings ranked the permeability of the microstructures as follows: acicular ferrite exhibited the lowest permeability, followed by bainite, while pearlite showed the highest. This can be explained by the discoveries of Thomas et al.^43^, who found that cementite in pearlite accelerates hydrogen permeation. Interfaces between different phases also influence hydrogen diffusion rates. Hesam et al.^44^ studied the mechanical properties of pipeline steels as a function of dissolved hydrogen in the microstructure and it was found that for a given dissolved hydrogen, steel with ferritic microstructure and smaller grain size showed lower reduction is elongation and toughness compared to the steels with higher amount of pearlite and larger grain size.

Binhan et al.^45^ introduce a novel strategy to improve hydrogen embrittlement resistance in high-strength steels by leveraging chemical heterogeneity within the material’s microstructure. They demonstrated that manganese-rich zones act as hydrogen traps and enhance crack resistance, effectively preventing hydrogen-induced damage without compromising strength or ductility. This approach broadens the scope for microstructure engineering to optimize materials for hydrogen environments.

Hagi^37^ studied the effect of the interface between cementite and ferrite on hydrogen diffusion in carbon steel (0–0.6 mass%) with spheroidized cementite-ferrite and pearlite-ferrite structures. The results showed that hydrogen diffusivity decreases with increasing carbon concentration, primarily due to the trapping effects of cementite-ferrite interfaces and dislocations. Additionally, the binding energy between a hydrogen atom and the cementite-ferrite interface is lower for planar cementite compared to spherical cementite. Tau et al.^38^ investigated the influence of pearlite/ferrite alignment on hydrogen permeation in AISI 4130 steel. They observed significant anisotropy in hydrogen diffusivity within the banded structure, with the highest diffusivity measured along the longitudinal (L) direction, followed by the transverse (T) direction, and the lowest in the through-thickness (S) direction. In contrast, the random ferrite/pearlite structure exhibited more uniform hydrogen diffusivity across all directions. Furthermore, the random structure showed higher diffusivity than the banded structure in both the transverse and through-thickness directions but lower diffusivity in the longitudinal direction. Pengwei et al.^46^ concluded that interfaces such as martensite/ferrite, martensite/austenite, and ferrite/austenite can act as reversible hydrogen traps that impede diffusion. Hence, additional investigations are required for a comprehensive understanding of hydrogen permeation behavior in relation to different microstructural features.

The role of ferrite/cementite interfaces in hydrogen trapping is complex and influenced by factors such as deformation and microstructural characteristics. Li et al.^47^ investigated the effect of deformation on hydrogen trapping in pearlitic steel. Scanning transmission electron microscopy and atom probe tomography analyses revealed that deformation led to strain localization at the ferrite/cementite interfaces, along with the formation of a high density of dislocations. This resulted in significant hydrogen segregation at the interfaces. Therefore, hydrogen trapping in deformed pearlitic steels is primarily attributed to the strained ferrite/cementite interfaces. Thermal desorption spectroscopy (TDS) studies by Kawakami et al.^48^ detected hydrogen desorption peaks associated with ferrite/cementite interfaces, indicating the presence of hydrogen trapping sites at these locations. The trap energy of these interfaces was also quantified, providing further evidence of their trapping role. Additionally, Mirzoev et al.^49^ used ab initio calculations and thermodynamic analysis to study hydrogen interaction with ferrite/cementite interfaces. They concluded that even thin lamellar ferrite/cementite structures, with interlamellar spacing below 0.1 μm, exhibit noticeable hydrogen trapping capability at temperatures below 400 K.

Hydrogen permeation and trapping in pipeline steels have significant implications for the integrity and long-term performance of natural gas infrastructure. While previous studies have examined hydrogen behavior in various steel types, limited research has systematically investigated the effects of microstructural evolution over different decades of steel production. This study addresses this gap by conducting electrochemical permeation experiments on three types of natural gas pipeline steels—modern, vintage, and legacy—installed in Canada during different eras. The primary objective of this research is to quantify key parameters, including the effective hydrogen diffusion coefficient (D_eff_), hydrogen sub-surface concentration (C_0R_), and hydrogen trap density (N_T_). By correlating these parameters with specific microstructural features such as grain size, dislocation density, and pearlite content, this study provides a comprehensive framework for understanding hydrogen behavior in pipeline steels. Unlike previous works that focus primarily on single-generation steels or qualitative analyses, this research introduces a comparative approach across different steel generations, offering new insights into the evolution of hydrogen susceptibility in pipeline materials.

The novelty of this study lies in the systematic evaluation of hydrogen trapping mechanisms through experimental data and schematic illustrations that delineate the interactions between hydrogen and different microstructural features. The research also uniquely integrates electrochemical permeation data from prior studies for comparative analysis, providing a broader context for the findings. These insights are critical for assessing the long-term reliability of pipeline steels and informing future material design strategies to mitigate hydrogen-related degradation. While this study focuses on hydrogen transport and trapping mechanisms, the authors’ ongoing research will further investigate the impact of hydrogen on the mechanical properties of pipeline steels, specifically ductility and fracture toughness. Overall, this work advances the fundamental understanding of hydrogen diffusion and trapping behaviors across different generations of pipeline steels, bridging the gap between scientific investigation and practical applications in pipeline integrity management.

## Results

### Microstructural analysis

**Fig. 1:**
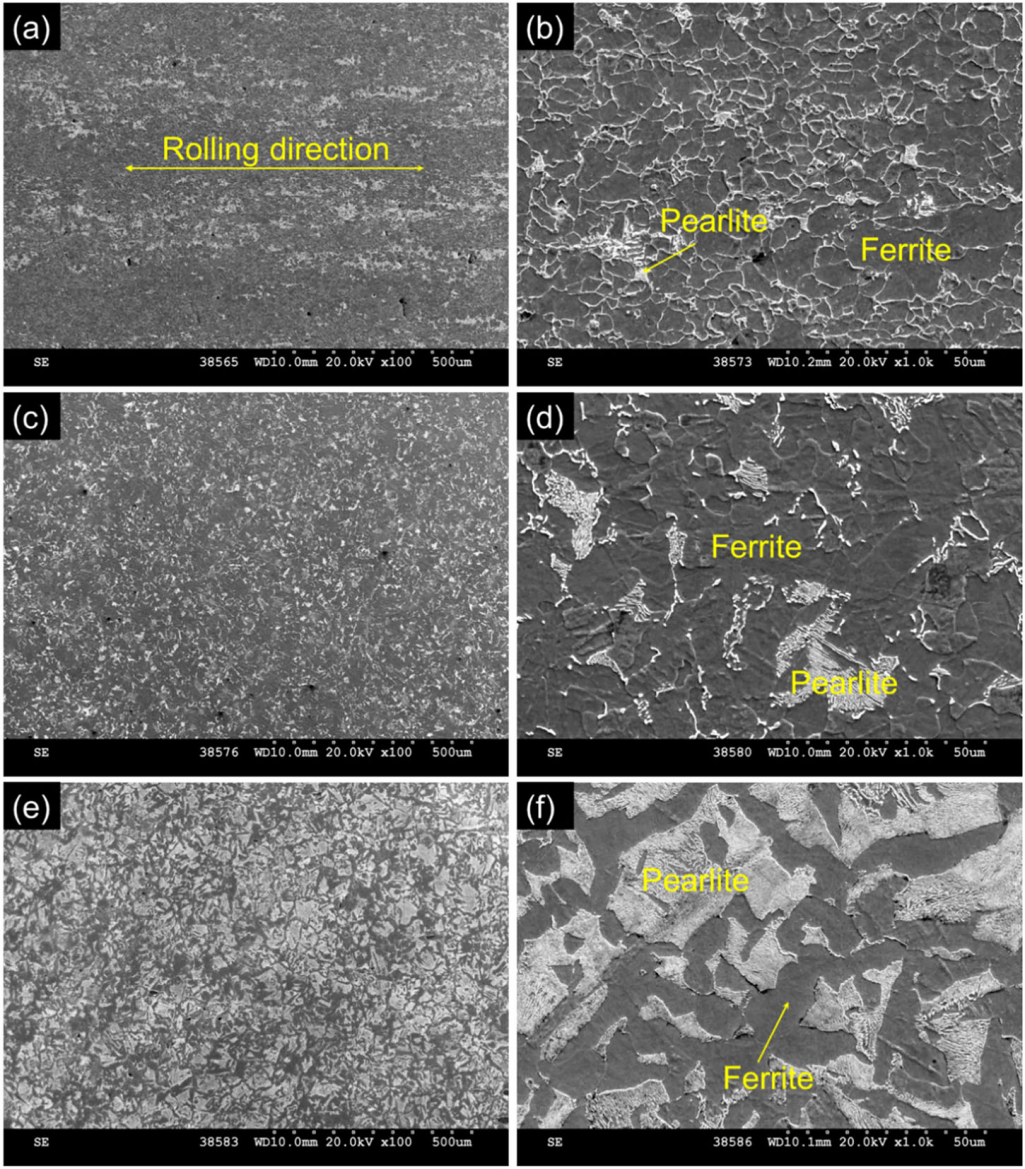
Microstructural comparison of modern, legacy, and vintage pipeline steels using SEM. Scanning electron micrographs captured at 20 kV for: **a**, **b** modern steel, **c**, **d** legacy steel, and **e**, **f** vintage steel. Images reveal differences in microstructure, including phase distribution.

Scanning electron microscopy (SEM) and optical microscopy were employed to examine the microstructures of the different steels after polishing to 1 µm and etching with 10% nital. The modern steel exhibited much finer grains and a higher ferrite content compared to the legacy and vintage steels, as shown in Fig. 1 and detailed in Table 1. This can be attributed to the strengthening mechanisms of modern pipeline steels, which rely on thermomechanical rolling and grain size refinement, rather than pearlite, to increase yield strength. Thermomechanical rolling also introduces significant anisotropy, with pearlite in modern steel forming bands along the rolling direction, as seen in Fig. 1a.

**Table 1:** Microhardness and grain size measurement

Material	Average grain size (µm)	Percentage of pearlite (%)	Overall hardness (HV_1kgf_)	Ferrite hardness (HV_25gf_)	Pearlite hardness (HV_25gf_)
Modern	15.4 ± 4.0	5.5 ± 1.9	224.8 ± 2.0	213.4 ± 13.7	242.6 ± 25.3
Legacy	59.5 ± 6.5	25.8 ± 3.7	148.4 ± 2.8	154.1 ± 4.5	173.9 ± 21.4
Vintage	88.0 ± 15.4	47.8 ± 5.8	202.4 ± 4.1	149.8 ± 18.5	175.7 ± 10.0

In contrast, Fig. 1c, d shows that the legacy steel contains more pearlite and larger grains than the modern steel, and it is more isotropic since it was not subjected to rolling. Although primarily composed of ferrite with pearlite inclusions, the legacy steel has a more coarse-grained structure. The vintage steel, shown in Fig. 1e, f, is predominantly pearlitic with very large, isotropic grains. At the time of its production, the primary strengthening mechanism was through carbon-induced pearlite, with less emphasis on grain size refinement, which is reflected in its microstructure.

**Fig. 2:**
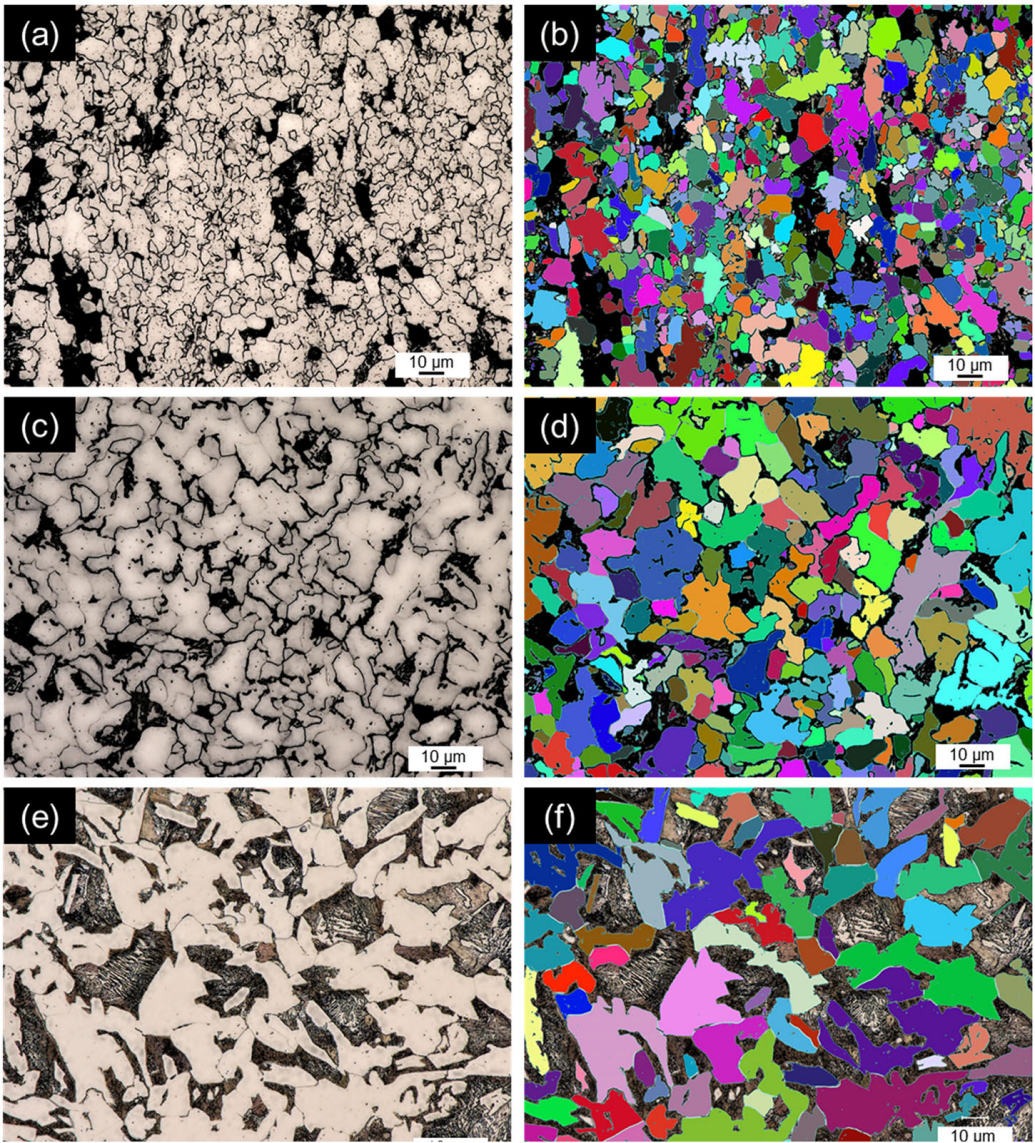
Grain characterization in pipeline steels using optical microscopy with and without threshold overlays. Optical micrographs at 1500× magnification showing grain size analysis for: **a**, **b** modern steel, **c**, **d** legacy steel, and **e**, **f** vintage steel. Images **b**, **d**, and **f** include threshold mask overlays used for automatic grain counting and sizing.

The optical micrographs in Fig. 2 were used for automatic grain size analysis, with contrast enhancement applied to distinguish between ferrite, pearlite, and grain boundaries more clearly. Keyence VHX software was employed to create a threshold mask overlay, detecting the bright regions and outlining ferrite grains, as the pearlite and grain boundaries appeared darker. The mask was manually adjusted to include low-contrast grain boundaries and the grain areas were tabulated to calculate the average grain size. The same process was repeated for pearlite by analyzing the dark regions. The grain size measurements are summarized in Table 1.

Vickers microhardness testing was performed on all three steels to measure overall hardness and the individual phase hardness of ferrite and pearlite, with results presented in Table 1. Legacy steel was found to be the softest, as it has relatively coarse grains but less pearlite than vintage steel, making it the least strengthened of the three. The hardness of both phases in modern steel is higher than that of the older steels, likely due to grain boundary strengthening.

### Electrochemical permeation

**Fig. 3:**
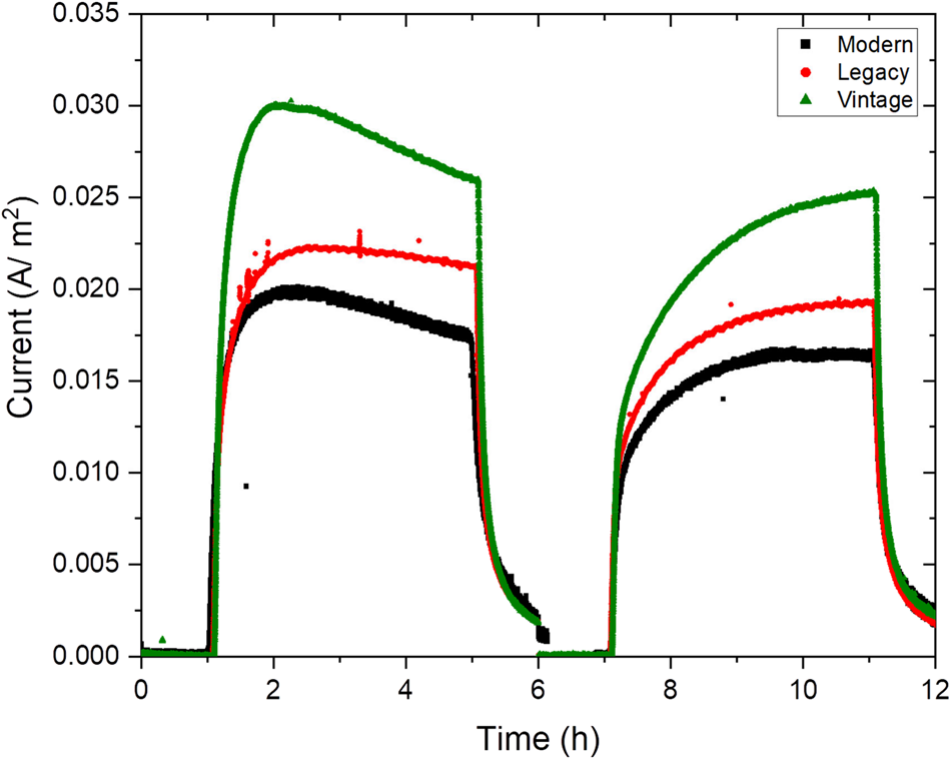
Electrochemical hydrogen permeation behavior across steel types. The curves depict hydrogen flux over time under identical test conditions, indicating material-dependent permeability for modern, legacy, and vintage steels.

The permeation current curve for the pipeline steels is shown in Fig. 3. Once the PSU was activated, the permeation current began to rise, eventually reaching a plateau. At this point, all reversible and irreversible hydrogen traps were saturated. When the PSU was turned off, the permeation current rapidly decreased.

**Fig. 4:**
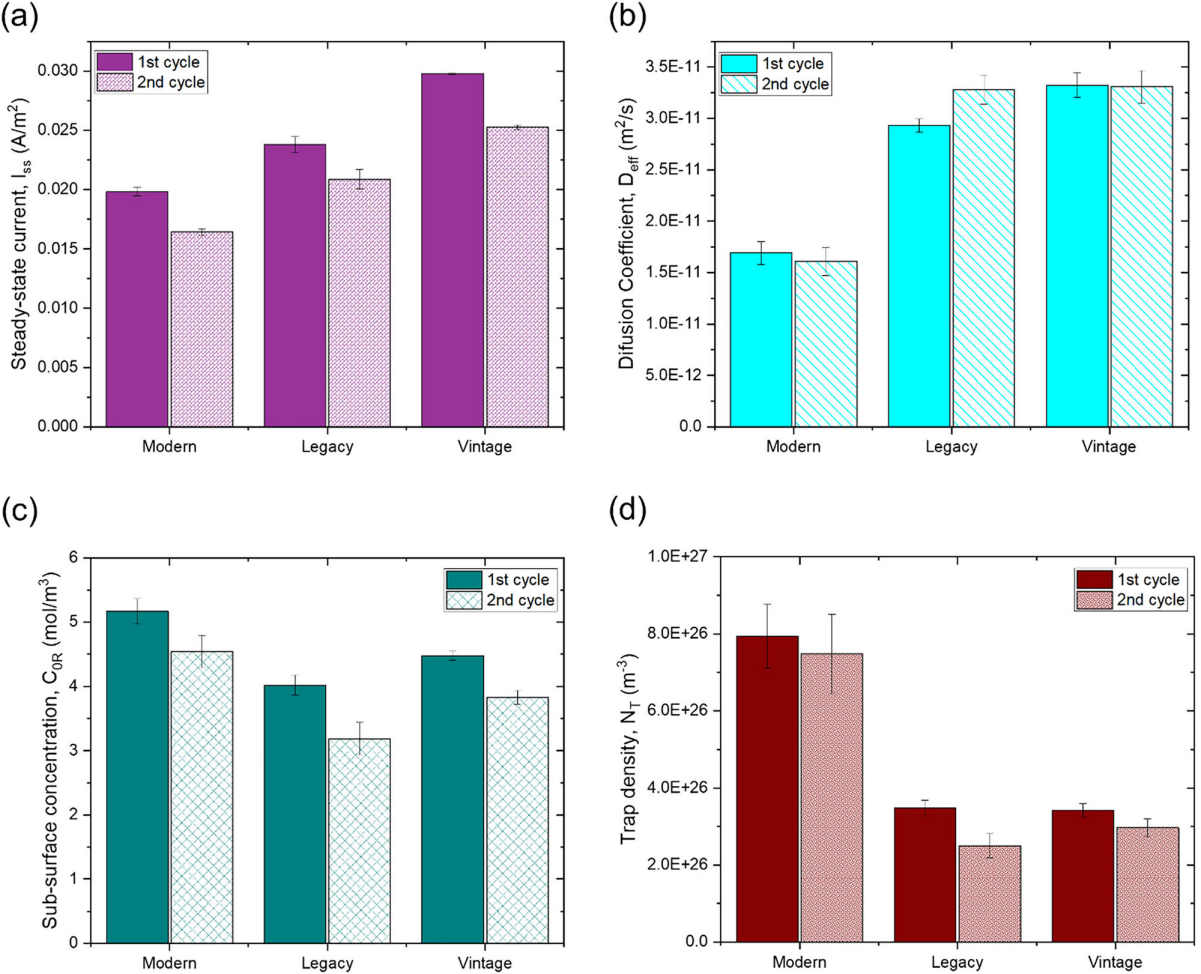
Comparison of key electrochemical hydrogen permeation parameters in different pipeline steels. Plots of: **a** steady-state permeation current (I_ss_), **b** effective diffusion coefficient (D_eff_), **c** sub-surface hydrogen concentration (C₀_R_), and **d** trap density (N_T_) for modern, legacy, and vintage steels. The data reflect distinct trapping and transport characteristics across steel grades.

Figure 4 presents the electrochemical permeation test results for the evaluated pipeline steels. The effective diffusivity (D_eff_), shown in Fig. 4b, was calculated using the time lag (t_lag_) or slope method, which provided more consistent results than the time breakthrough (t_b_) method.

As illustrated, the vintage pipeline steel exhibited the highest steady-state permeation current (I_ss_) and effective diffusivity (D_eff_), while the modern steel recorded the lowest values, with the legacy steel showing intermediate results. These differences can be attributed to variations in dislocation density, grain boundary effects, and overall steel microstructure. The grain boundaries are believed to act more as hydrogen traps than as diffusion pathways, potentially decreasing hydrogen permeation rates. In this study, modern steel demonstrated a lower hydrogen permeation rate of 1.69 × 10^−11^ m^2^/s compared to the other steels. However, it trapped almost twice as much hydrogen as the older steels during the first cycle, as shown in Fig. 4d, resulting in the highest sub-surface hydrogen concentration, 5.17 mol/m^3^ (Fig. 4c).

**Fig. 5:**
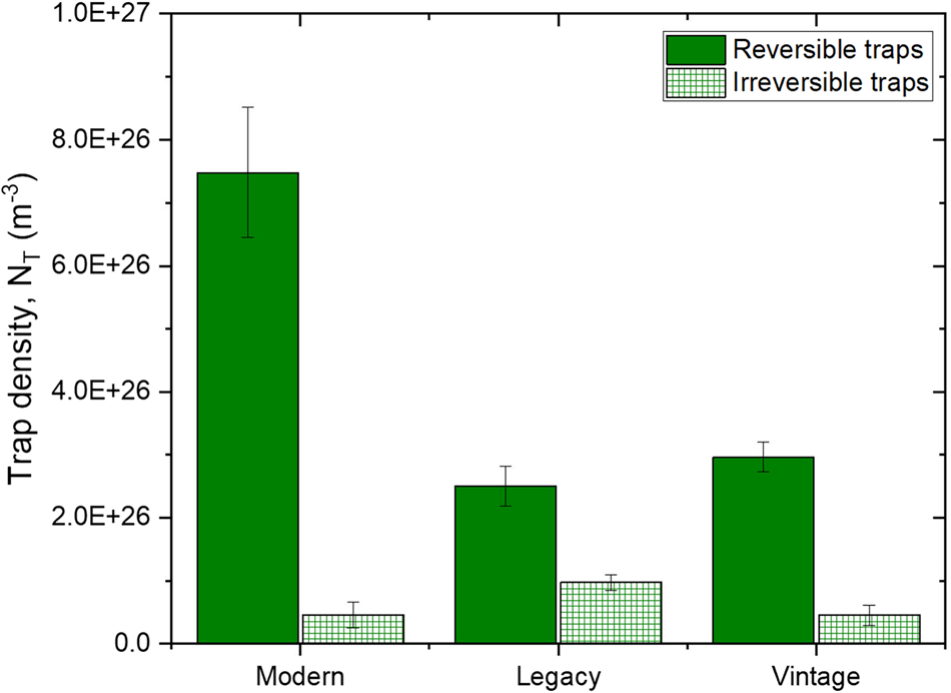
Distribution of reversible and irreversible hydrogen traps in pipeline steels. Bar chart comparing reversible and irreversible trap densities in modern, legacy, and vintage steels, indicating different hydrogen retention behaviors.

Figure 5 depicts the reversible and irreversible hydrogen trapping behavior for the different pipeline steels. Modern steel showed significantly higher reversible trapping at 7.48 × 10^26^ m^−3^, compared to the legacy and vintage steels, which had similar densities of 2.50 × 10^26^ m^−3^ and 2.97 × 10²⁶ m^−3^, respectively. This occurs because dislocations typically serve as reversible hydrogen traps due to their relatively low binding energy with hydrogen atoms^50^. In contrast, modern steel had the lowest irreversible trapping density at 0.45 × 10²⁶ m^−3^, close to vintage steel at 0.46 × 10²⁶ m^−3^. Legacy steel exhibited a higher irreversible trap density of 0.97 × 10²⁶ m^−3^.

Pressouyre et al.^51^ studied the influence of hydrogen trapping on embrittlement and developed a model to predict the effects of microstructural features interacting with hydrogen. They proposed that a critical localized concentration of hydrogen is necessary for embrittlement to occur. Based on this model, traps are classified as “good” or “bad” depending on their ability to either delay the time required to reach the critical concentration or to disperse hydrogen, preventing localized accumulation. Reversible traps, due to their lower binding energy, allow hydrogen to move more freely, potentially leading to increased localized concentration in certain site and promote embrittlement. In contrast, irreversible traps, with higher binding and activation energy, are more effective at containing hydrogen at a specific site.

**Fig. 6:**
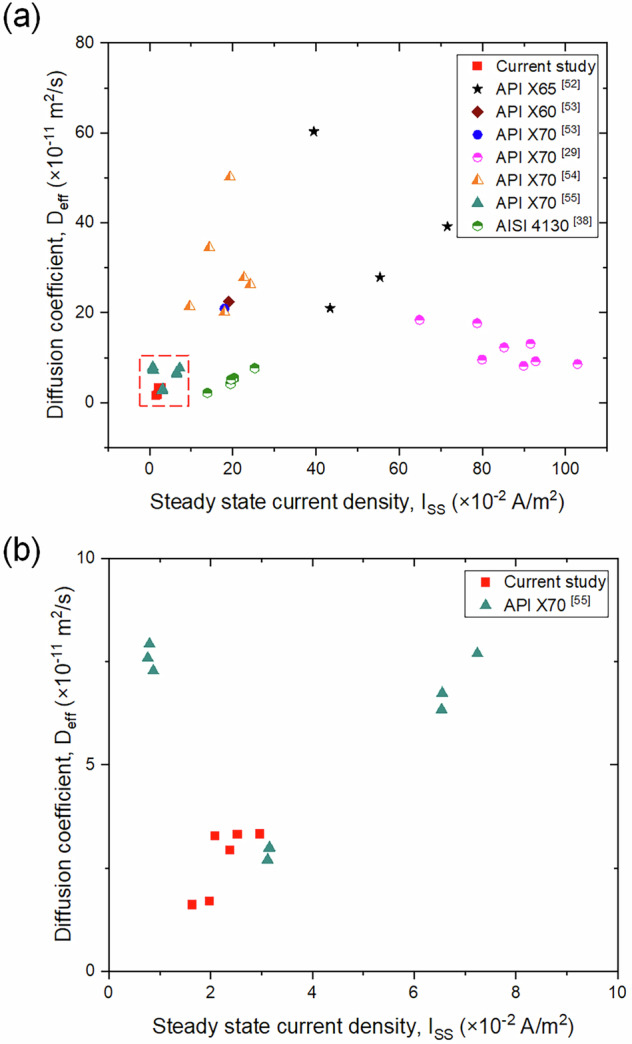
Relationship between steady-state current and diffusion coefficient in various steels. **a** Scatter plot of I_ss_ versus D_eff_ across multiple steel samples, and **b** Magnified view of the clustered region in (**a**) to highlight differences in low-diffusivity materials.

Figure 6 presents the relationship between steady-state current density (I_ss_) and the effective diffusion coefficient (D_eff_) for various steels. In Fig. 6a, the data points from the current study (in red) are plotted alongside data from the literature (in black)^29,38,52–55^. The plot shows a wide variation in D_eff_ values for different steels, with some steels exhibiting an increase in D_eff_ as I_ss_ increases, while most do not follow a specific trend. These differences in behavior are attributed to variations in microstructural features, inclusions and defects, charging conditions, and the thickness of test coupons.

Figure 6b provides a magnified view of the lower I_ss_ range, highlighting the current study’s data points. The increasing effective diffusion coefficient with respect to steady-state current suggests a concentration-dependent diffusion^56^. Hagi et al.^57^, in their study on dislocation trapping of hydrogen in iron, concluded that the activation energy for diffusion in a normal lattice is mass-dependent. However, the trap site density and binding energy are, within experimental error, independent of the mass of the diffusing atoms. It is important to note that in this study, the charging conditions for all three evaluated steels are constant, meaning the amount of hydrogen produced on the entry side is also constant. Modern steel, which contains the highest trap density, exhibited the lowest steady-state current and diffusion coefficient compared to vintage and legacy steel. This is primarily due to trapped hydrogen and its interaction with the hydrogen diffusing through the steel. Future experimental and modeling work is needed to account for this dependence.

### Dislocation density of pipeline steels

**Fig. 7:**
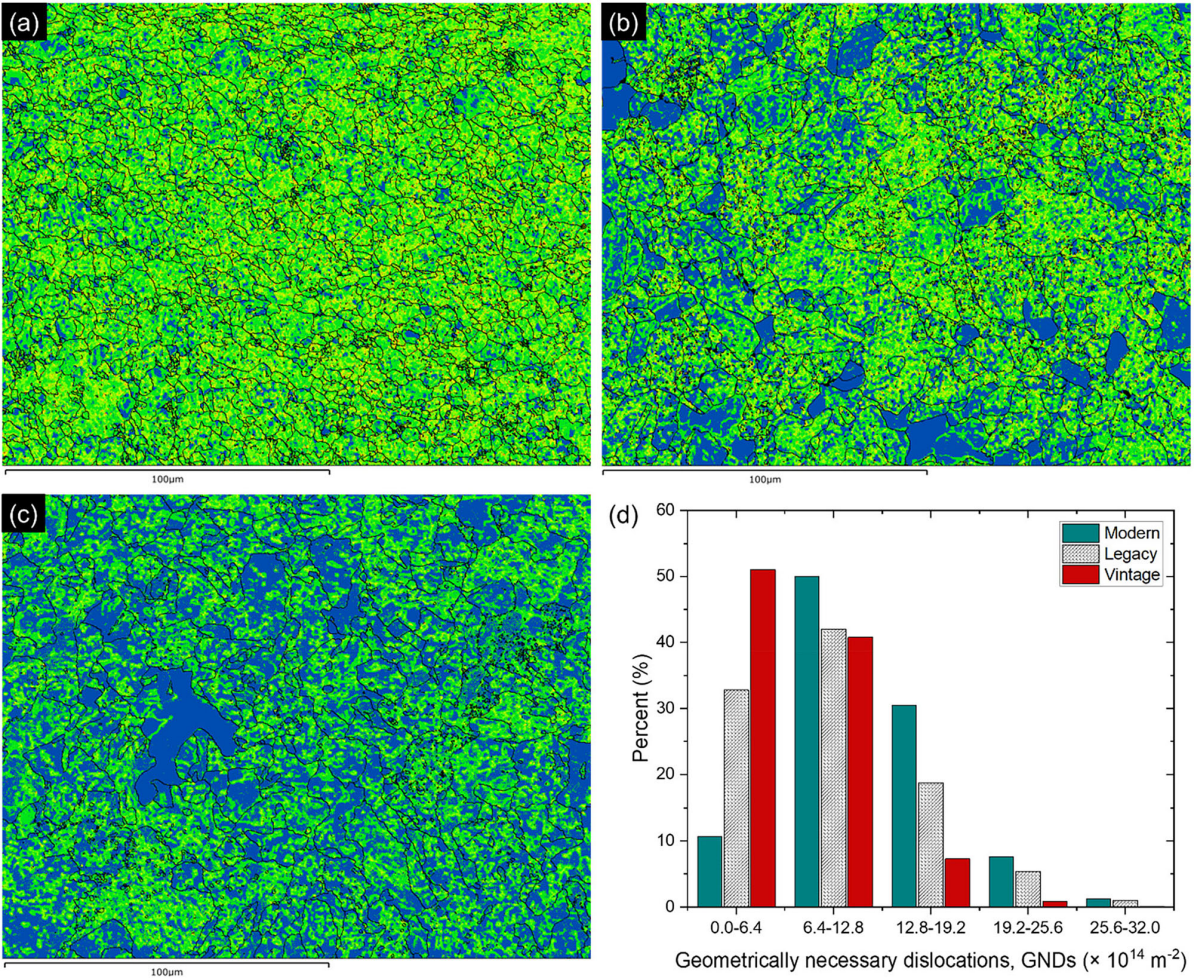
Geometrically necessary dislocation density (GND) distribution in pipeline steels. **a**–**c** GND density maps obtained via electron backscatter diffraction (EBSD) for modern, legacy, and vintage steels, respectively. **d** Bar graph summarizing average GND densities for the three steel types.

Figure 7a–c shows the geometrically necessary dislocation density (GND) maps for modern steel, legacy steel, and vintage steel, respectively. The color gradient used in the maps indicates different levels of GND density, helping to visualize the dislocation density within each steel type, with the following ranges: blue (0.0–6.4 × 10^14 ^m^−2^), bright green (6.4–12.8 × 10^14 ^m^−2^), light green (12.8–19.2 × 10^14 ^m^−2^), yellow (19.2–25.6 × 10^14 ^m^−2^), and red (25.6–32.0 × 10^14 ^m^−2^).

Figure 7d is a histogram that illustrates the distribution of GND densities across these three types of steels. The x-axis represents GND densities and the y-axis shows the percentage of each density range present within the samples. It is evident that vintage steel (red) predominantly falls within the lowest GND density range (0–6.4 × 10^14 ^m^−2^) indicating the lowest amount of dislocations, whereas modern steel shows a more even distribution across the mid and high ranges, and legacy steel displays a significant presence in both low and mid-range GND densities.

**Fig. 8:**
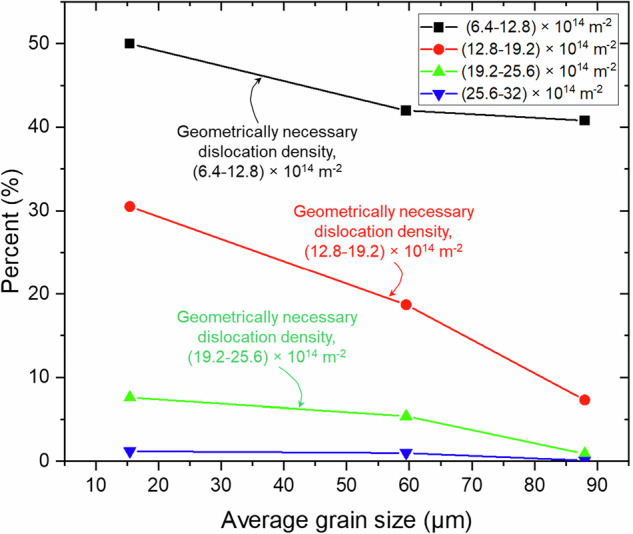
Correlation between average grain size and geometrically necessary dislocation density. Scatter plot showing the inverse relationship between grain size and GND density in pipeline steels. Data suggest a microstructural basis for dislocation-mediated hydrogen trapping.

Figure 8 illustrates the relationship between average grain size and the percentage of geometrically necessary dislocation density (GND) for the three types of pipeline steels. As grain size increases, the percentage of GND density decreases across all density ranges. The lowest GND density range (6.4–12.8 × 10^14 ^m^−2^) shows the highest percentage, particularly at smaller grain sizes, and steadily declines as grain size increases. The next GND density range (12.8–19.2 × 10^14 ^m^−2^) also exhibits a significant percentage, which reduces drastically with larger grain sizes. The higher GND density ranges (19.2–25.6 × 10^14 ^m^−2^ and 25.6–32.0 × 10^14 ^m^−2^) show lower percentages overall, with minimal values at larger grain sizes. This indicates that finer grains in the pipeline steels tend to have higher GND densities, and as the grain size increases, the density of GNDs decreases, particularly in the lower density categories. This result aligns with the microstructure of modern steel, as illustrated in Fig. 1a, b, where thermomechanical rolling and grain size refinement were performed to increase the yield strength of the materials, eventually inducing dislocation during the process.

### Factors effecting the permeation behaviour

**Fig. 9:**
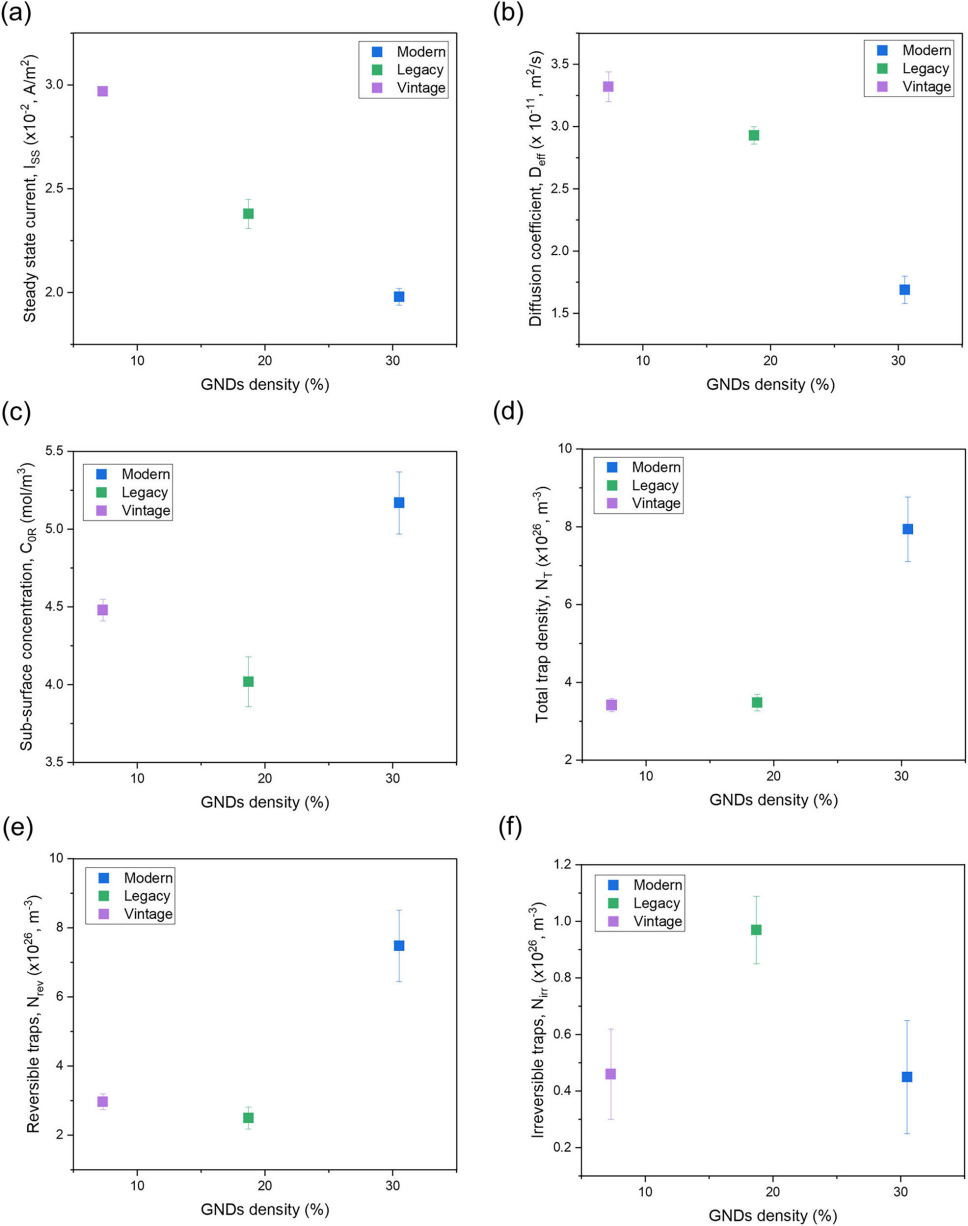
Influence of dislocation density on electrochemical hydrogen permeation characteristics. Plots of geometrically necessary dislocation density (GNDs, in the range of 12.8–19.2 × 1014 m^−2^) versus: **a** steady-state current, **b** diffusion coefficient, **c** sub-surface concentration, **d** total trap density, **e** reversible trap density, and **f** irreversible trap density.

Figure 9 presents the relationships between geometrically necessary dislocation densities (GNDs) and various parameters obtained from electrochemical hydrogen permeation tests conducted on modern, legacy, and vintage pipeline steels. The x-axis represents the GND density percent (%) for the range of 12.8–19.2 × 10¹⁴ m^−2^, while the y-axis displays different parameters relevant to hydrogen permeation behavior.

Figure 9a illustrates the relationship between GND density and the steady-state current (I_ss_). As shown, the steady-state current generally decreases with an increase in GND density. Specifically, a reduction of approximately 20–33% in the steady-state current is observed for dislocation densities of 30% and 19%, respectively, compared to a 7% dislocation density. A similar behavior is observed for the diffusion coefficient (D_eff_), where a decrease is seen with an increase in GND density, indicating that higher dislocation densities may hinder hydrogen diffusion in these materials (Fig. 9b). In this case, reductions of about 12% and 49% are observed for dislocation densities of 30% and 19%, respectively, compared to a 7% dislocation density. In this study. the effect of dislocation density is more pronounced for the diffusion coefficient than for the steady-state current.

Figure 9c shows the sub-surface concentration of hydrogen (C_0R_) relative to GND density. In theory, the sub-surface concentration of hydrogen should increase with increasing dislocation density, as dislocations act as trap sites for hydrogen. In this study, an increase in sub-surface hydrogen concentration is observed with increasing GND density, from vintage to modern steel. However, legacy steel, which has a higher dislocation density, exhibits a lower sub-surface hydrogen concentration than vintage steel. This suggests that other microstructural factors may also influence the sub-surface concentration of hydrogen.

Figure 9d–f represents the total trap density (N_T_), reversible traps (N_rev_), and irreversible traps (N_irr_) relative to GND density, respectively. Both total trap density (N_T_) and reversible traps (N_rev_) generally increase with increasing dislocation density, indicating a greater capacity for trapping and releasing hydrogen reversibly in steels with high GND density. Conversely, irreversible traps (N_irr_), which depend on binding energy, do not follow a specific trend with dislocation density, as other microstructural features such as micro-cracks, voids, and alloy precipitates may primarily be responsible for these traps.

**Fig. 10:**
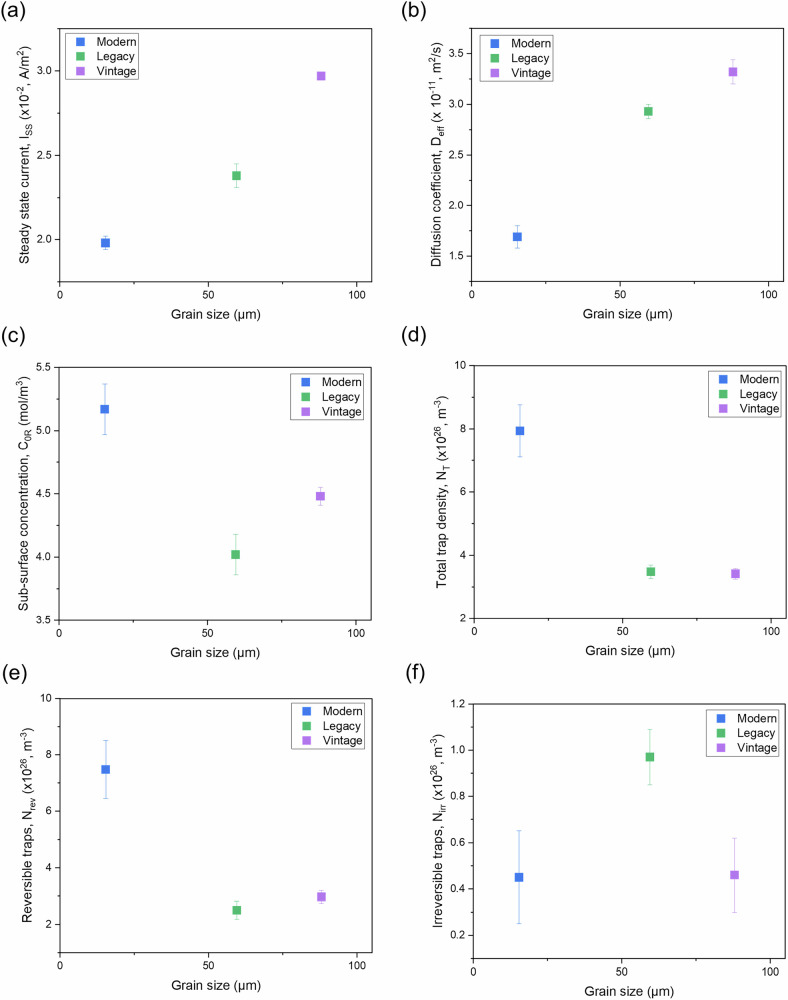
Effect of grain size on the electrochemical hydrogen permeation behaviour of pipeline steels. Grain size plotted against: **a** steady-state current, **b** diffusion coefficient, **c** sub-surface hydrogen concentration, **d** total trap density, **e** reversible traps, and **f** irreversible traps.

Figure 10 illustrates the relationships between grain size and various parameters obtained from hydrogen permeation tests. The x-axis represents grain size, while the y-axis shows different hydrogen permeation parameters.

Figure 10a displays the relationship between grain size and steady-state current (I_ss_). The results indicate that the steady-state current generally increases with grain size. Specifically, a 50% increase in steady-state current is observed as grain size increases from 15 μm to 88 μm. This suggests that larger grains, with fewer grain boundaries, offer less resistance to hydrogen permeation, leading to higher steady-state current.

The diffusion coefficient (D_eff_) as a function of grain size is illustrated in Fig. 10b. The diffusion coefficient also increases with grain size, supporting the hypothesis that larger grains facilitate easier hydrogen diffusion due to fewer trapping sites like grain boundaries. The study reveals a 73% increase in the diffusion coefficient as grain size grows from 15 μm to 60 μm, with a more modest 13% increase as grain size expands from 60 μm to 88 μm.

Figure 10c presents a comparison of sub-surface hydrogen concentration (C_0R_) with respect to grain size. Modern steel, with its finer grains and higher dislocation densities, exhibits the highest sub-surface hydrogen concentration, while legacy steel shows the lowest. Notably, despite its larger grain size, vintage steel exhibits a higher sub-surface hydrogen concentration than legacy steel. It is important to note that the sub-surface layer thickness, typically in the nanometer range, and grain size may not significantly impact the sub-surface hydrogen concentration. Instead, other microstructural features, such as dislocations, active diffusion mechanisms, and available interstitial sites near the sub-surface, likely play a more dominant role in influencing hydrogen concentration.

The relationship between grain size and different types of hydrogen traps: total trap density (N_T_), reversible traps (N_rev_), and irreversible traps (N_irr_) are illustrated in Fig. 10d–f, respectively. Both total trap density (N_T_) and reversible traps (N_rev_) decrease with increasing grain size, indicating that smaller grains with more grain boundaries are more effective at trapping hydrogen. This trend is consistent with modern steel, which has the smallest grain size and the highest density of both reversible and total traps. In contrast, irreversible traps (N_irr_) do not show a clear trend with grain size, suggesting that factors other than grain size, such as micro-cracks, voids, or alloy precipitates, play a more significant role in determining the density of irreversible traps.

**Fig. 11:**
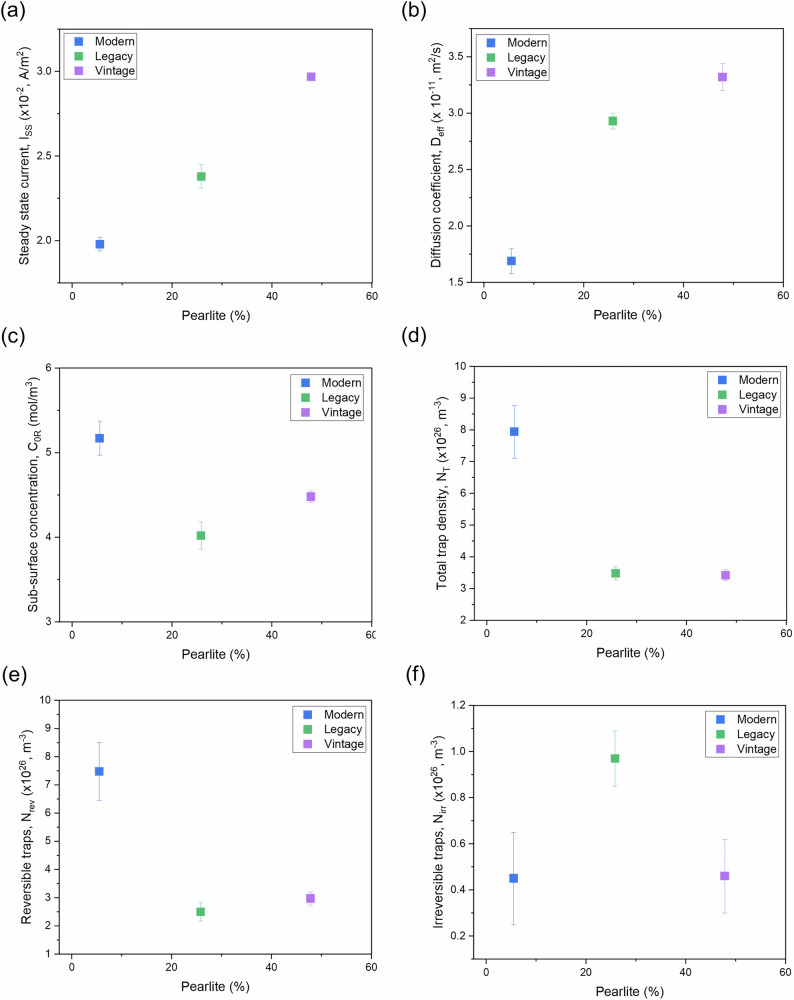
Effect of pearlite content on hydrogen permeation properties of pipeline steels. Pearlite volume fraction (%) plotted against: **a** steady-state current, **b** diffusion coefficient, **c** sub-surface hydrogen concentration, **d** total trap density, **e** reversible traps, and **f** irreversible traps. Pearlite content influences hydrogen trapping and permeation parameters.

Figure 11 illustrates the relationship between pearlite content and various parameters related to hydrogen permeation in pipeline steels.

As shown in Fig. 11a, the steady-state current (I_ss_) increases with increasing pearlite content. The vintage steel, with the highest pearlite percentage, exhibits the highest steady-state current while modern steel, with the lowest pearlite content, shows the lowest steady-state current. An increase in pearlite content from 6% to 26% and from 26% to 48% resulted in approximately 20% and 25% increases in steady-state current, respectively.

The diffusion coefficient (D_eff_) shows a similar trend (Fig. 11b), where vintage steel, containing the highest pearlite, has the highest diffusion coefficient, indicating faster hydrogen transport. In contrast, modern steel, with minimal pearlite, exhibits the lowest diffusion coefficient. Tau et al.^38^ investigated how ferrite/pearlite alignment affects hydrogen permeation in AISI 4130 steel. They concluded that pearlitic blocks hinder hydrogen diffusion, resulting in significant anisotropy in the banded structure. In a random ferrite/pearlite arrangement, hydrogen diffuses more uniformly in all directions, leading to higher diffusivity compared to the transverse and through-thickness directions of the banded structure. However, diffusivity in the random structure is lower than in the longitudinal direction of the banded structure, where hydrogen moves freely along the ferrite bands. Lee et al.^41^ also investigated the hydrogen embrittlement of AISI 4130 steel with an alternating ferrite/pearlite banded structure. They found that the effective hydrogen diffusivity was lowest in the through-face direction, nearly an order of magnitude less than in other directions. While the longitudinal and transverse sections had similar diffusivities, they exhibited different permeabilities, with the longitudinal direction showing a higher hydrogen permeation flux.

Similar to the effect of grain size, the sub-surface concentration of hydrogen (C_0R_) does not follow a straightforward trend, as shown in Fig. 11c. In general, the sub-surface concentration of hydrogen decreases as pearlite content increases. However, vintage steel, despite having higher pearlite content than legacy steel, exhibits a higher sub-surface concentration of hydrogen than legacy steel.

Figure 11d–f illustrates the trapping behavior of pipeline steels concerning pearlite content. The total trap density (N_T_) and reversible traps (N_rev_) follow similar trends, decreasing with increasing pearlite content. Modern steel, which contains the least pearlite, has the highest total and reversible trap density, indicating more sites for hydrogen trapping. This observation aligns with the understanding that lower pearlite content, particularly in acicular ferrite-rich microstructures, results in a higher number of trapping sites, slowing down hydrogen permeation. While legacy steel exhibits the fewest reversible traps, vintage steel, which has the highest pearlite content, exhibits the lowest total trap density, whereas legacy steel shows the highest number of irreversible traps (N_irr_) among the evaluated steels.

These results are consistent with previous studies^42,43^ that demonstrate how pearlite accelerates hydrogen permeation due to the presence of cementite, which acts as a pathway for hydrogen diffusion.

## Discussion

**Fig. 12:**
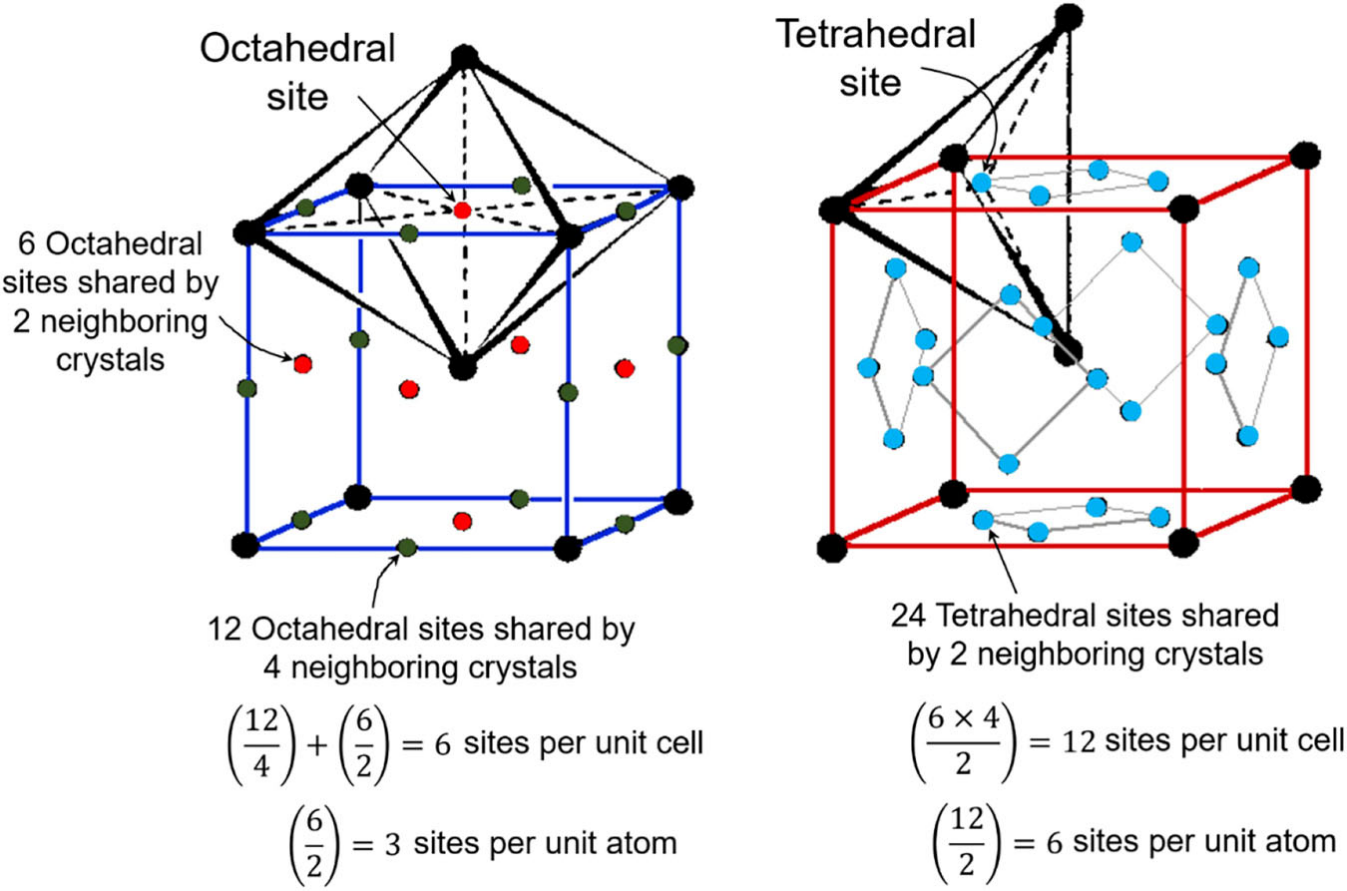
Hydrogen occupancy sites in body-centered cubic (BCC) iron crystal structure. Schematic diagram illustrating octahedral and tetrahedral interstitial sites in BCC iron. These are potential locations for hydrogen atoms within the crystal lattice.

According to classical diffusion theory, hydrogen atoms move by hopping from one interstitial lattice site to another. Figure 12 illustrates the octahedral and tetrahedral lattice sites of BCC crystal such as ferrite. As shown, there are three octahedral and six tetrahedral lattice sites per unit atom. Generally, the radius of a tetrahedral site in BCC is approximately twice that of an octahedral site at ambient temperature. Consequently, tetrahedral lattice sites are the preferred interstitial sites, particularly at low temperatures, as they can more readily accommodate hydrogen atoms. In contrast, octahedral sites are the preferred interstitial sites for metals containing FCC crystal structures such as austenitic stainless, while for HCP crystal the preferred sites are tetrahedral at ambient temperatures^58^. FCC and HCP crystal structures comprises of one octahedral and two tetrahedral sites per atom. On the other hand, in BCC crystals, each atom contains three octahedral and six tetrahedral sites.

In BCC crystals, possible jumps between tetrahedral sites occur either directly between a tetrahedral site and four nearest-neighbor tetrahedral sites along the 〈110〉 directions or across octahedral sites to two next-nearest neighbors in the 〈100〉 directions. On the other hand, in FCC crystals, the close-packed atomic rows prevent direct jumps from one octahedral site to another in the 〈110〉 directions. As a result, hydrogen migration paths involve intermediate jumps in the 〈111〉 directions to the nearest neighbor tetrahedral sites, followed by jumps from tetrahedral to octahedral sites. An octahedral site in an FCC crystal is surrounded by eight nearest neighbor tetrahedral sites in the 〈111〉 directions and twelve octahedral sites in the 〈110〉 directions. A tetrahedral site, in turn, is surrounded by four octahedral sites^59^.

The spacing between nearest-neighbor tetrahedral sites in BCC crystals is roughly half that of the nearest-neighbor octahedral sites in FCC crystals^60^, which results in significantly lower activation energies for hydrogen diffusion in BCC structures compared to FCC structures^61,62^. According to Wipf^63^, the typical distance between nearest-neighbor interstitial sites in a BCC lattice is approximately 0.11 nm (tetrahedral sites), whereas in an FCC lattice, the typical distance is about 0.18 nm. A greater separation distance generally results in higher activation energy for hydrogen diffusion. This accounts for the higher hydrogen diffusion rates observed in BCC iron (ferrite) compared to austenitic stainless steel, which has an FCC crystal structure^60,64^. Additionally, Grabert et al.^65^ and Schober et al.^66^ have suggested that at low temperatures, quantum tunneling between adjacent interstitial sites in BCC metals becomes an important diffusion mechanism, allowing hydrogen atoms to move without overcoming the energy barrier.

Diffusion of hydrogen requires overcoming the diffusion activation energy (EₐD), which is the energy needed for a hydrogen atom to move from one lattice site to another. The hydrogen atom moves between interstitial sites by jumping over energy barriers, specifically the saddle point energy (E_s_), which is the energy barrier that hydrogen atoms must overcome to transition from a lattice site to a trap site. The figure highlights two types of trapping sites: reversible and irreversible. In a reversible trap, the hydrogen atom can escape back to a lattice site if it acquires sufficient trap activation energy (EₐT), which is the energy required for the hydrogen atom to leave the trap site and return to a normal lattice site. However, if the atom becomes trapped in an irreversible site, the trap binding energy (E_B_), which represents the energy binding the hydrogen atom to the trap site, is so significant that the atom cannot overcome this energy barrier, effectively immobilizing it. It is important to note that there is no definitive binding energy threshold that clearly distinguishes reversible traps from irreversible ones. Essentially, as the binding energy increases, the likelihood of an atom escaping from a trap reduces gradually until it becomes negligible. Naturally, with a rise in temperature, this probability increases, leading to a shorter residence time of hydrogen in the trap and an overall increase in effective diffusivity^67^. From a thermodynamic perspective, if the activation energy exceeds the trap binding energy of an irreversible site, the trapped atom can escape. Irreversible trapping is considered in some systems to have no direct impact on cracking. Saenz et al.^68^ and Jeklih et al.^55^ demonstrated that deep trapping, or irreversible trapping, does not individually affect cracking in 13Cr martensitic stainless steels and in carbon steel. Although there are some exceptions, generally deeper trap sites tend to be filled with hydrogen during processing and have limited influence on diffusivity^68^. The light red circles in this figure illustrate possible future positions of the hydrogen atom, showing potential transitions between different energy states. The figure emphasizes the interplay between the various energy terms: saddle point energy (E_s_), diffusion activation energy (EₐD), trap binding energy (E_B_), and trap activation energy (EₐT), which collectively govern the movement and trapping behavior of hydrogen within the material. Activation energy for different types of traps is tabulated in Table 2.

**Table 2:** Activation energy and type of hydrogen traps for different microstructural features in steel

Microstructural features	Activation energy (KJ/mol)	Type of trap	References
Grain boundary	9.0–49.0	Reversible	^30,33,89–91^
Cementite-ferrite interface	11.0–18.0	Reversible	^89,92^
Dislocation strain field	12.0–27.0	Reversible	^33,89,90,93^
Cementite interlaces	18.4	Reversible	^33^
Dislocation	26.0–33.9	Reversible	^33,94^
Microvoids in cold worked iron	35.2–40.3	Reversible or irreversible	^33,89,90,95^
Microvoids	40.3	Reversible or irreversible	^95,96^
Austenite/Ferrite interface	37.0–44.0	Reversible or irreversible	^97,98^
Interface of Fe-oxide	43.0–62.0	Reversible or irreversible	^90,92,99^
Dislocation (bulk)	60.0	Reversible or irreversible	^98^
Interface of MnS	64.0–72.3	Reversible or irreversible	^90,92,95,100^
Interface of Al_2_O_3_	71.0–79.0	Reversible or irreversible	^90,92,95^
Interface of TiC	87.0	Irreversible	^90,95,101^

The rate at which hydrogen atoms hop between interstitial lattice sites and the extent to which they become transiently trapped at various microstructural trap sites govern the diffusion of hydrogen in a metal. The potential well at these trap sites is deeper than at interstitial lattice sites, causing the hydrogen atom to remain at the trap site longer. In case of irreversible trap sites, the potential well is particularly deep and the probability of the hydrogen atom escaping from the trap site becomes effectively zero. The influence of trapping on diffusion is determined by the density, distribution, and depth of the potential wells associated with these trap sites.

**Fig. 13:**
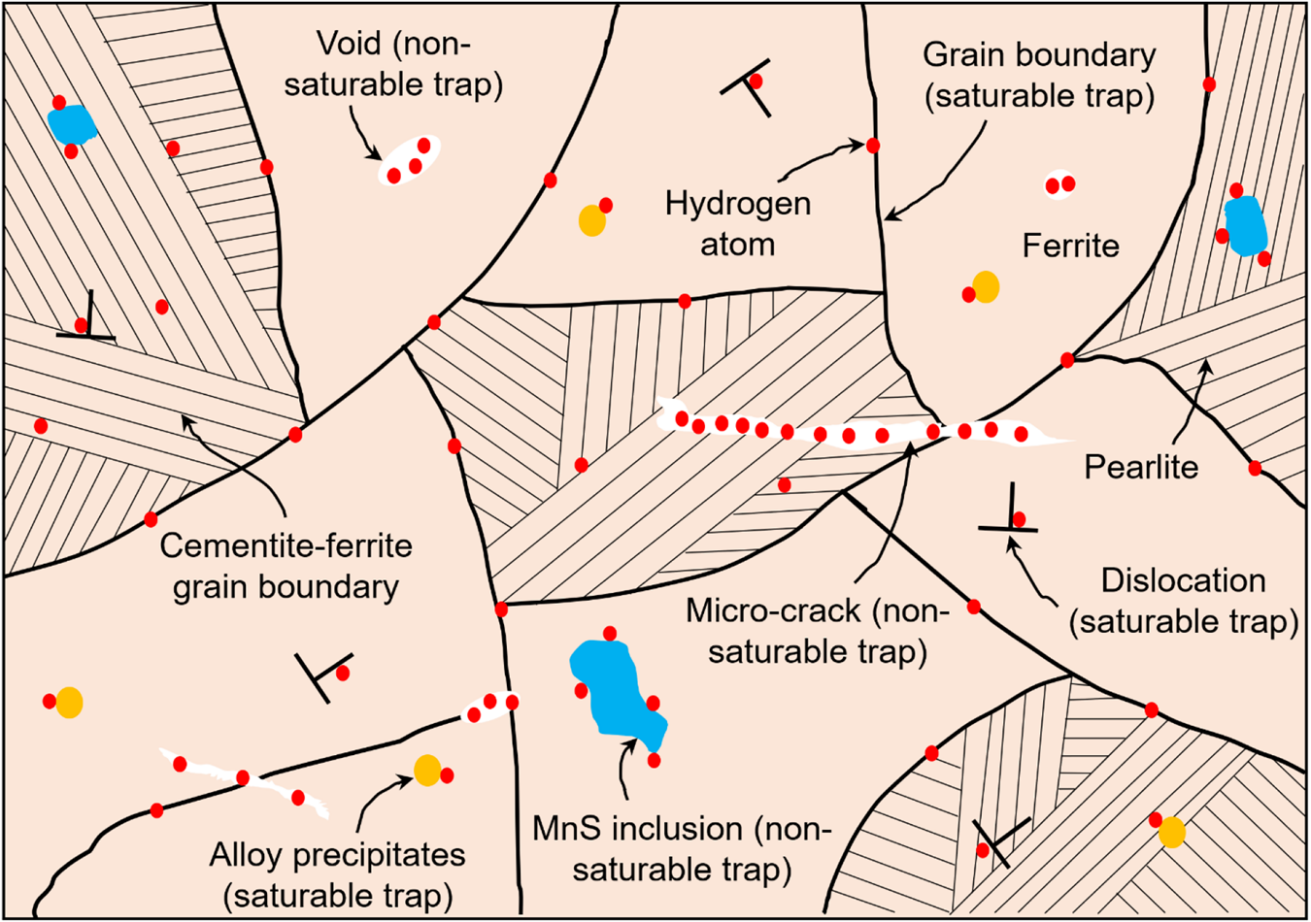
Illustration of hydrogen trapping mechanisms within steel microstructures. Schematic representation of hydrogen interactions with various trapping features such as dislocations, grain boundaries, inclusions, and precipitates in steel.

In practice, metals can exhibit a spectrum of trap sites, each characterized by a specific binding energy. However, typically one or two types of traps tend to dominate and significantly influence the effective diffusivity at a given temperature. These microstructural traps include dislocations, grain boundaries, interfaces between the matrix, phase boundaries, and inclusions or particles, solute atoms, and vacancies. The hand-drawn schematic microstructure of steel (Fig. 13) highlights the interaction between hydrogen atoms and various microstructural features. Depending on their capacity for hydrogen, traps can be classified as saturable or non-saturable. Saturable traps have a limited capacity for hydrogen, while non-saturable traps can increase their capacity with increased hydrogen gas pressure.

Grain boundaries, particularly those between ferrite and pearlite, act as saturable traps for hydrogen. The accumulation of hydrogen at these boundaries can weaken cohesive forces, potentially leading to intergranular fracture under stress. Similarly, dislocations serve as saturable traps that attract hydrogen atoms. High dislocation density can enhance the likelihood of hydrogen-induced cracking, especially in stressed regions.

Hydrogen trapping at voids and micro-cracks is a unique case, where the void represents a dynamic sink for molecular hydrogen, sometimes described as non-saturable. Voids formed at interfaces, such as MnS inclusions, can also act as dynamic traps, leading to phenomena such as near-surface blistering or internal void growth. The accumulation of hydrogen in these features can lead to the growth of voids and cracks, increasing the risk of fracture, particularly under cyclic loading.

Alloy precipitates and second-phase particles, such as carbides or nitrides, also act as saturable traps, providing nucleation sites for hydrogen atoms and influencing their distribution within the steel. These interactions can impact the steel’s toughness and resistance to cracking.

It is interesting to note that once trap sites are saturated with hydrogen, the trapped atoms can interfere with diffusion by reducing the number of available sites for hydrogen movement (hopping), leading to slower diffusion (as discussed in the following section). However, under certain conditions, such as changes in temperature or mechanical loading, traps with relatively low binding energy may release hydrogen back into the steel matrix, contributing to higher permeation.

During electrochemical hydrogen permeation, the steady-state current (I_ss_) represents the constant current when hydrogen permeation through a metal membrane reaches equilibrium—where the rate of hydrogen entering the metal equals the rate of its exit. The steady-state current is directly related to the amount of hydrogen permeating through the metal. Experimental data indicate that when hydrogen is blended with natural gas, vintage steel experiences the highest hydrogen permeation, followed by legacy steel, while modern steel exhibits the highest resistance to hydrogen permeation.

It was found that the steady-state current increases with grain size and pearlite content in the microstructure, while it decreases with dislocation density. This is likely because both grain boundaries and dislocations act as hydrogen traps, inhibiting hydrogen permeation, whereas pearlite facilitates permeation, as evidenced by previous studies.

**Fig. 14:**
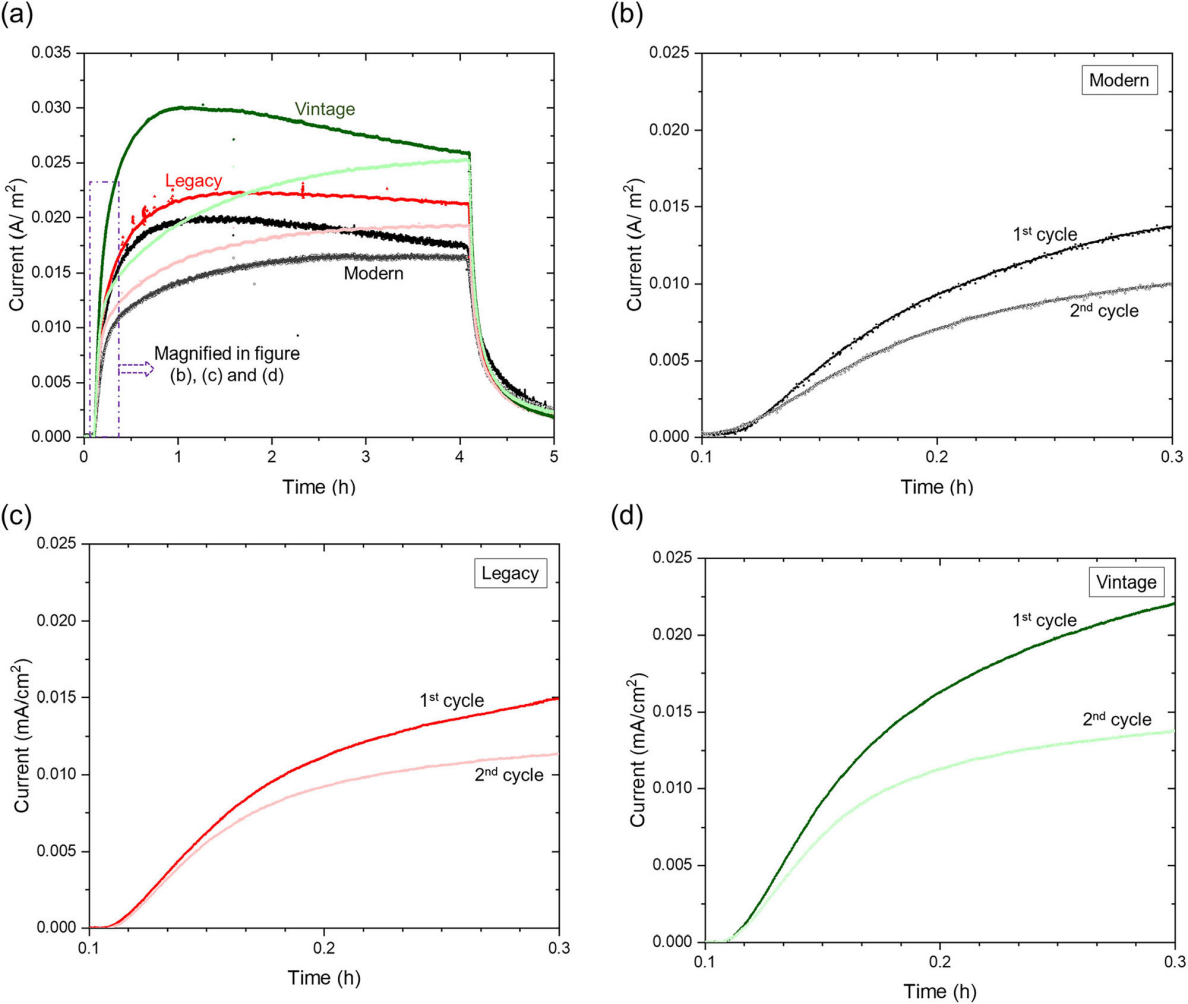
Comparison of electrochemical permeation profiles across charging cycles and steel types. **a** Overlay of the 1st and 2nd permeation curves. **b** Magnified initial permeation region for modern steel, **c** legacy steel, and **d** vintage steel. Differences in transient behavior reflect material-dependent hydrogen diffusion dynamics.

Figure 14 illustrates the electrochemical permeation profile, comparing the first and second permeation cycles of pipeline steels. In Fig. 14a, the first and second permeation cycles of pipeline steels are superimposed, with dark colors representing the 1^st^ cycle and lighter colors representing the 2^nd^ cycle. The figure shows differences in the initial stages of permeation. Figure 14b–d provide a magnified view of the initial permeation curves for modern, legacy, and vintage steel, respectively. Initially, within about 10 min, the diffusion for both cycles is faster, as indicated by the steeper slope of the current response. As time progresses (after 10 min), the rate slows down, evident from the decreasing slope. In the second cycle, diffusion is slower compared to the first, likely due to trapped hydrogen in irreversible trap sites within the steel. These trapped hydrogen atoms may interfere with diffusion by reducing the number of available sites for hydrogen movement (hopping), leading to slower diffusion in the second cycle. Although diffusion in the second cycle is slower, the diffusion coefficient for legacy and vintage steel is slightly higher or similar to that of the first cycle (Fig. 4b). This is likely because the steady-state current is 12% and 15% lower in the 2^nd^ cycle for legacy and vintage steel, respectively, requiring slightly less time to reach 63% of the steady-state current. In addition, the slope changes from steep to gradual after the initial break-in period.

**Fig. 15:**
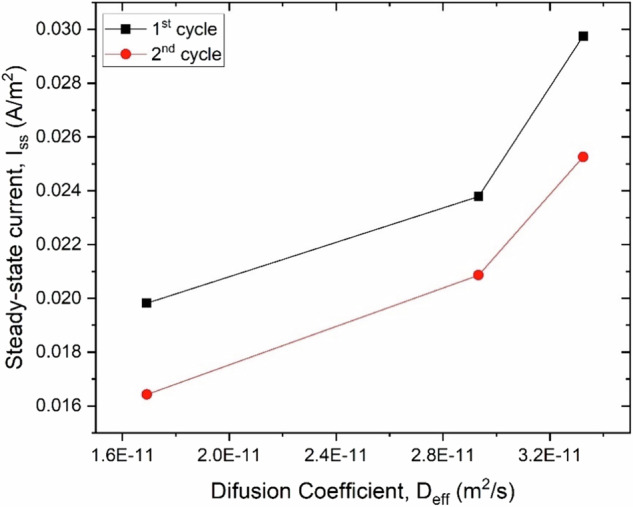
Variation of diffusion coefficient with steady-state current across charging cycles. Scatter plot comparing D_eff_ versus I_ss_ for both 1^st^ and 2^nd^ electrochemical charging cycles. Trends highlight the impact of charging history on permeation properties.

In this study, the hydrogen diffusion coefficient (D_eff_) increases with grain size and pearlite content and decreases with dislocation density, following a similar trend as the steady-state current. Figure 15 presents the relationship between the diffusion coefficient and steady-state current for the 1^st^ and 2^nd^ cycles. The figure shows that as the diffusion coefficient increases, the steady-state current also increases for both cycles, suggesting that materials with higher diffusion coefficients allow more hydrogen to permeate. However, after the first cycle, when the material becomes saturated with hydrogen, the second cycle shows a lower steady-state current, indicating less hydrogen permeation.

The sub-surface lattice hydrogen concentration (C_0R_) is a key parameter for concentration-driven diffusion and also serves as an indicator of the environmental severity^67^. According to Protopopoff et al.^59^ hydrogen atoms at entry sites are closer to sub-surface metal atoms than those in the bulk, leading to slightly higher hydrogen concentrations at the sub-surface. In this study, sub-surface hydrogen concentration generally increases with dislocation density and grain boundary area.

**Fig. 16:**
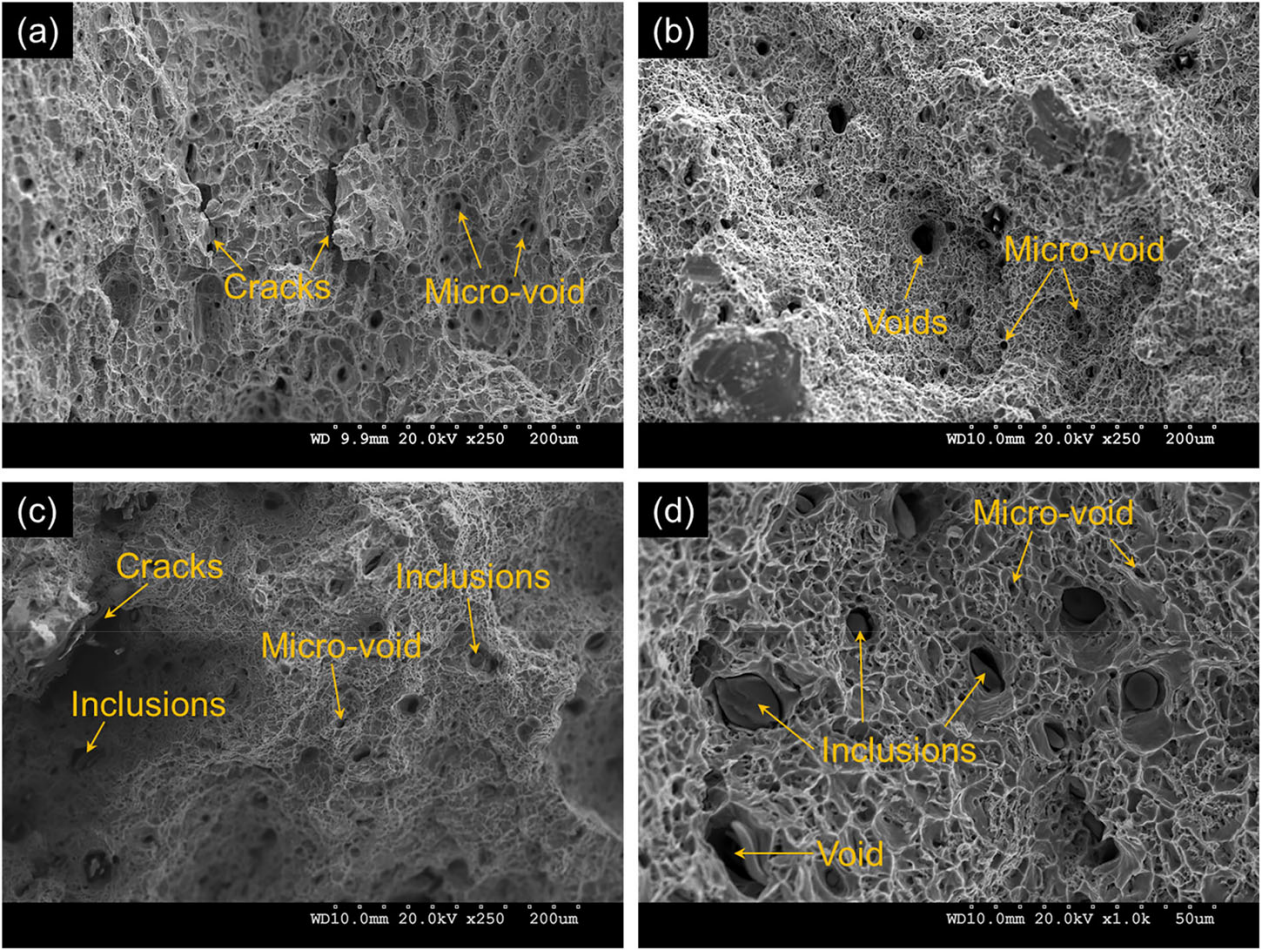
Fractographic features of modern, legacy, and vintage pipeline steels. Fracture surfaces from tensile-tested samples showing: **a** modern steel, **b** legacy steel, **c** vintage steel, and **d** magnified view of legacy steel. Images illustrate voids, microcracks, and inclusion-induced fracture mechanisms.

As illustrated in Fig. 6a, the diffusion coefficient and permeation current for different steels exhibit significant scatter. This variability is attributed to differences in microstructural features, inclusions and defects, charging conditions, and the thickness of test coupons. In addition to the influence of different microstructural phases such as pearlite, ferrite, bainite, and martensite, inclusions and defects also play a critical role in hydrogen diffusion and permeation behavior, as evidenced by the permeation test data in this study. To further illustrate this, Fig. 16 presents the fracture surfaces of modern, legacy, and vintage steels from the authors’ ongoing research. This comparison is relevant to the discussion of hydrogen trapping, as voids and inclusions act as irreversible hydrogen traps. The figure clearly demonstrates that different steels contain varying amounts of voids and defects. Consequently, drawing definitive conclusions about the trapping behavior of grain boundaries and cementite-ferrite lamella is challenging, as voids and defects interfere with these mechanisms.

Modern steel exhibits the lowest amount of voids and defects, whereas legacy steel contains a higher density of voids and inclusions (e.g., MnS) compared to vintage steel. These microstructural features are directly linked to hydrogen trapping behavior, as voids, micro-cracks, and inclusions such as MnS and alloy precipitates have high trap activation energy, leading to increased irreversible trapping in legacy steel.

In this study, reversible traps constitute approximately 94%, 72%, and 87% of the total traps in modern, legacy, and vintage steels, respectively. Consequently, total trap density follows a similar trend to reversible traps. In general, both total and reversible trap densities decrease with increasing grain size and pearlite content but increase with dislocation density. While bulk dislocations exhibit high activation energy (~60 kJ/mol), irreversible traps do not follow a specific trend with dislocation density, grain size, or pearlite content.

In summary, dislocation density, grain size, pearlite-ferrite content, and defects (porosity, voids, inclusions, micro-cracks) collectively influence hydrogen permeation and embrittlement susceptibility in pipeline steels. Modern steel, while offering the highest resistance to hydrogen permeation, may experience more significant reductions in mechanical properties due to increased trapped hydrogen in the sub-surface.

In this study, electrochemical tests were conducted on three pipeline steels: modern (installed in 2016, CSA Grade 448/API 5L X65), legacy (installed in 1975, CSA Grade 290/API 5L X42), and vintage (installed in 1954, CSA Grade 359/API 5L X52). The key findings are as follows:

- Modern steel exhibited the highest resistance to hydrogen permeation, followed by legacy steel, while vintage steel showed the highest hydrogen permeation rates. This indicates that modern steel is more resistant to hydrogen permeation compared to older steels.
- The steady-state current (I_ss_) and diffusion coefficient (D_eff_) were found to increase with grain size and pearlite content, while they decreased with dislocation density. This suggests that grain boundaries and dislocations act as hydrogen traps, inhibiting hydrogen permeation, whereas pearlite facilitates permeation.
- Modern steel exhibited significantly higher reversible trap density compared to legacy and vintage steels. Conversely, legacy steel had the highest density of irreversible traps, likely due to the presence of more voids and inclusions such as MnS.
- The sub-surface hydrogen concentration (C_0R_) generally increased with dislocation density and grain boundary area. Modern steel, with the smallest grain size, showed the highest sub-surface hydrogen concentration.
- Defects such as porosity, voids, inclusions, and micro-cracks significantly influence hydrogen permeation and embrittlement susceptibility. Modern steel had the least amount of porosity and defects, while legacy steel contained more voids and inclusions, accounting for its higher irreversible trap density.

This study highlights the importance of understanding microstructural features and their impact on hydrogen permeation and trapping behavior to mitigate hydrogen embrittlement in pipeline steels. Based on the findings from current study, it can be concluded that while modern steel offers the highest resistance to hydrogen permeation, it may experience greater reductions in mechanical properties due to increased trapped hydrogen in the sub-surface.

## Methods

### Materials characterization

In this study, material samples were collected from three different operational pipelines in Canada. The installation years of the pipeline materials were 2016, 1975, and 1954. In North America, the U.S. Pipeline and Hazardous Materials Safety Administration considers any pipeline installed before 1970 to be “vintage”^69^. The pipeline steels used in this study are referred to as ‘Modern’, which is CSA Grade 448, equivalent to API 5L X65, installed in 2016; ‘Legacy’, which is CSA Grade 290, equivalent to API 5L X42, installed in 1975; and ‘Vintage’, which is CSA Grade 359, equivalent to API 5L X52, installed in 1954.

Key differences between vintage, legacy and modern pipeline steel primarily lie in their chemical composition and manufacturing processes^9^. Vintage and legacy steel contains higher carbon content (0.2–0.4 wt%) and lower levels of alloying elements, resulting in a microstructure of polygonal ferrite and pearlite, which impacts toughness. Modern steel, however, focuses on reduced carbon content and increased alloying elements such as Nb, V, and Ti, leading to a microstructure of acicular and polygonal ferrite, and bainite, contributing to finer grains and improved toughness. Manufacturing advancements from open-hearth furnaces to processes such as electric arc furnaces, vacuum degassing and thermomechanical rolling enhance steel purity, microstructure, and mechanical properties^70^. Consequently, modern pipeline steel exhibits superior resistance to fracture, corrosion, and fatigue^71^.

The chemical composition of the pipeline steels was obtained using Optical Emission Spectrometry (OES) analysis (Table 3). Relative to modern steel, both legacy and vintage steels have a higher carbon content and fewer alloying elements such as Mn, Si, Ni, Cr, Mo, etc. This reflects the current trend in steel production to decrease carbon content while increasing the presence of alloying elements. Due to their elevated carbon content, the microstructures of legacy and vintage steels typically consist of polygonal ferrite and pearlite. On the other hand, the microstructure of modern steel encompasses acicular ferrite, polygonal ferrite, and possibly bainite. Modern steel also demonstrates a more refined grain structure^72^. Pearlite content increased proportionally to an increase in carbon content as expected according to the Fe-C metastable phase diagram. The percentage of pearlite and ferrite is calculated based on the carbon concentration and iron-carbon binary phase diagram (Table 4)^73^.

**Table 3:** Nominal composition of Modern, Legacy, and Vintage pipeline steels

Material	Nominal composition (wt. %)											
	C	Mn	P	S	Si	Cu	Ni	Cr	Mo	V	B	Fe
Modern	0.052	1.44	0.010	0.003	0.212	0.168	0.123	0.040	0.135	0.029	0.0003	Bal
Legacy	0.186	0.79	0.005	0.015	0.002	0.024	0.023	0.030	0.003	0.002	0.0001	Bal
Vintage	0.280	1.07	0.010	0.023	0.034	0.207	0.081	0.032	0.009	0.002	0.0001	Bal

**Table 4:** Calculated percentage of pearlite and ferrite

Pipeline steels	Phases	
	Ferrite	Pearlite
Modern	95.9%	4.1%
Legacy	77.8%	22.2%
Vintage	65.0%	35.0%

The microstructure and grain size of the steels was evaluated by cross sectioning a piece of the pipeline steel, followed by hot mounting in bakelite resin, then plane grinding at 220 grit and fine grinding and polishing at 9 µm, 3 µm, and 1 µm. The mounted and polished steels were then etched in 10% nital before imaging with optical microscopy (Keyence VHX-7000) and Scanning Electron Microscopy (SEM) (High-Resolution Quanta FEG 650 SEM Thermo Fisher Scientific Inc.). Grain size analysis was performed using the built in Keyence software with High Dynamic Range (HDR) contrast enhancement to highlight grain boundaries for automatic grain size measurement with manual adjustments to detected grains.

### Electrochemical permeation test

**Fig. 17:**
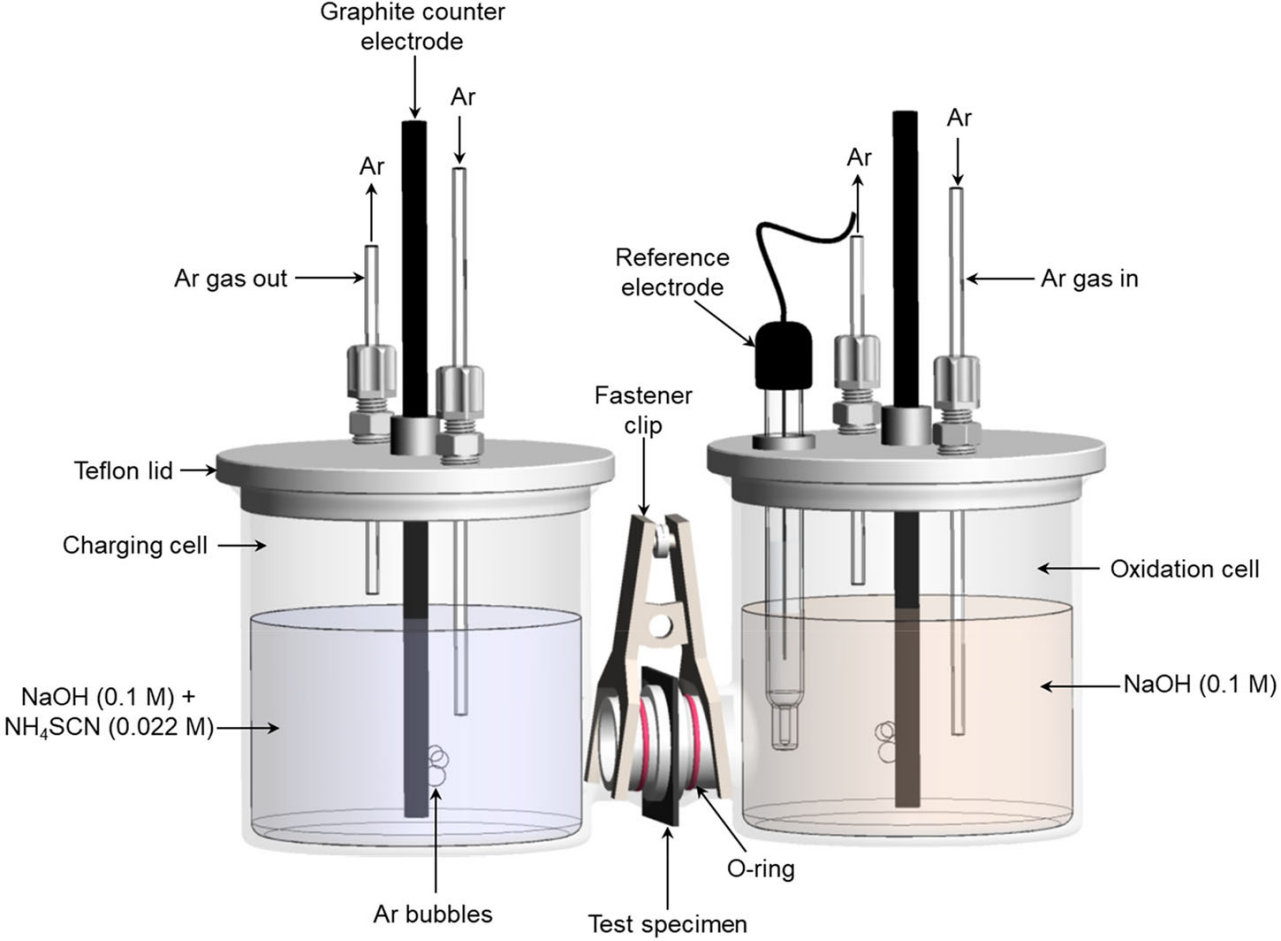
Schematic of the Devanathan–Stachurski cell used for electrochemical hydrogen permeation testing. This dual-cell setup design used to measure hydrogen permeation. The charging and detection cells are separated by the test specimen. The design allows for controlled hydrogen charging on one side and anodic detection on the other side.

Electrochemical permeation tests are used to determine the diffusion and trapping behavior of hydrogen in steel. In this paper, electrochemical permeation tests were performed in compliance with ASTM G148 using the Devanathan–Stachurski cell (D-S cell)^74^. Figure 17 illustrates the schematic of the experimental setup used in this study^75^.

**Fig. 18:**
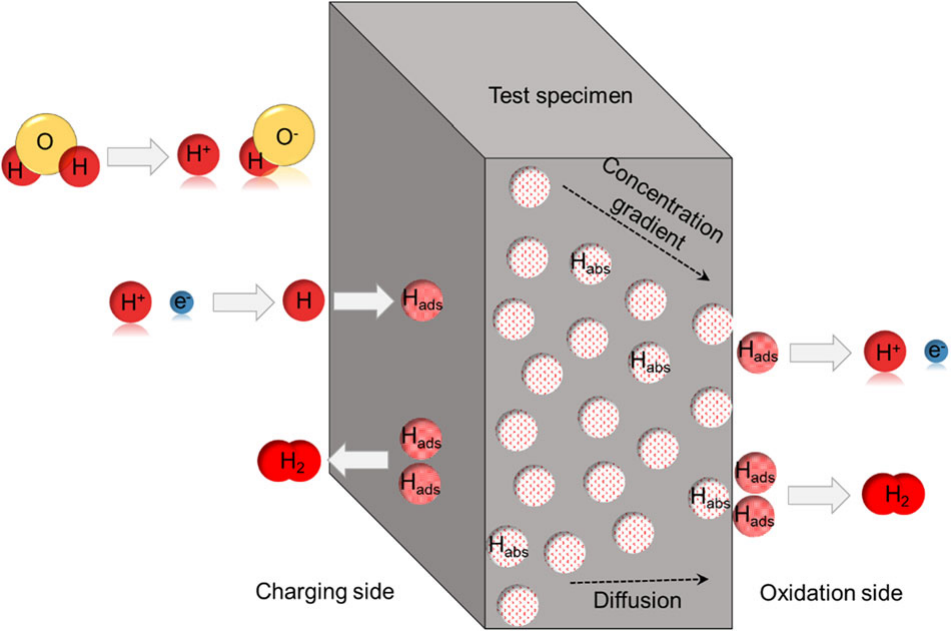
Illustration of the hydrogen permeation in steel using electrochemical methods. Hydrogen atoms generated on the charging side diffuse through the steel and are oxidized at the detection side, where the permeation current is measured.

Figure 18 illustrates a schematic representation of the electrochemical hydrogen permeation process through a test specimen. On the charging side, water (H₂O) is split into hydrogen ions (H⁺) and hydroxide ions (OH^−^). The hydrogen ions (H⁺) gain electrons, turning into hydrogen atoms (H), which then physically adsorb onto the surface of the test specimen. Some of the adsorbed hydrogen atoms (H_ads_) recombine to form hydrogen gas (H₂). To prevent this recombination and encourage hydrogen absorption, recombination poisons are added. This helps enhance the amount of hydrogen absorbed into the material. Once the hydrogen atoms adsorb on the surface, they are chemically absorbed (H_abs_) into the test specimen. Due to the concentration gradient between the charging and oxidation sides, hydrogen atoms diffuse through the material. On the oxidation side, the process is reversed. Hydrogen atoms (H_ads_) that reach the oxidation side lose electrons, producing hydrogen ions (H⁺) and an oxidation current that is measured by the potentiostat.

For the electrochemical permeation tests, all specimens were cut to dimensions of 0.5 mm × 35 mm × 35 mm. Each specimen was then ground using 600 grit SiC paper (USA). The specimens were cleaned using an ultrasonic cleaner, starting with a mixture of soap and deionized (DI) water (1:7 ratio), followed by DI water (18.2 MΩ), acetone, and finally methanol. The samples were then dried with compressed air.

The background current was measured to avoid interference and allow any hydrogen present in the steel from specimen preparation to diffuse out. The background current was measured over 8 h until it reached less than 250 nA∙cm^−2^. Once the background current was established, the hydrogen charging step began. In the charging cell, 150 mL of 0.1 M NaOH and 0.022 M ammonium thiocyanate (NH₄SCN) were added as recombination poison to enhance hydrogen absorption^76^.

Argon gas was bubbled in the charging side at a flow rate of 1 bubble per second to reduce oxygen presence in the electrolyte. A graphite rod was added to the charging cell and connected to a power supply unit (PSU) anode lead, while the cathode lead of the PSU was connected to the specimen. The PSU was then turned on to supply a constant current of 10 mA to the specimen, initiating the hydrogen charging procedure. When the PSU was activated, hydrogen atoms were generated in the charging cell, creating a concentration gradient between the two sides of the steel sample, causing hydrogen atoms to permeate toward the oxidation side.

As hydrogen atoms diffused into the oxidation cell, they lost electrons, producing the oxidation current, which was measured by the potentiostat. When the oxidation current plateaued after 4 h, the PSU was turned off, and hydrogen discharge began. The permeation process was repeated once under identical charging conditions. The objective of this repetition was to facilitate a second round of electrochemical permeation, where the sample had fully saturated irreversible traps. The pH of the charging side remained relatively stable throughout the cycles, indicating the stability of the electrolyte during the charging period. For modern and legacy steel, the pH varied slightly, measuring 13.41 before the 1^st^ cycle, 13.42 before the 2^nd^ cycle, and 13.44 after the 2^nd^ cycle. For vintage steel, the pH was 13.56 before the 1^st^ cycle, 13.50 before the 2^nd^, and 13.46 after the 2^nd^ cycle. Similarly, for legacy steel, the pH was 13.47, 13.46, and 13.45, respectively.

The mechanism behind electrochemical permeation can be conceptualized as the Volmer-Tafel-Heyrovsky reaction. Given the alkaline test environment in this study, the chemical reactions can be expressed as^77^:

1 $${H}_{2}O+M+{e}^{-}\to M{H}_{{ads}}+{{OH}}^{-}$$

2 $$2M{H}_{{ads}}\to {2M+H}_{2}$$

3 $$M{H}_{{ads}}+{H}_{2}O+{e}^{-}\to {H}_{2}+{{OH}}^{-}+M$$

4 $${{MH}}_{{ads}}\longleftrightarrow {{MH}}_{{abs}}$$

In Eq. (1), the process of chemisorption on the steel surface is detailed, a process referred to as the Volmer reaction. The Volmer reaction is predominantly observed when the overpotential remains at a lower level, which is a consequence of scant atomic hydrogen presence on the steel surface^78^. $M{H}_{{ads}}$ can recombine to form molecular hydrogen, as shown in Eq. (2), known as the Tafel reaction. In Eq. (3), the Heyrovsky reaction, $M{H}_{{ads}}$ has the potential to produce gaseous hydrogen under conditions of heightened overpotential, which is typically a result of an elevated surface occupancy by hydrogen atoms^78^. Simultaneously with the processes outlined in Eqs. (2) and (3), the adsorbed atomic hydrogen diffuses into the steel’s sub-surface layer, Eq. (4)^79^.

Following Eq. (4), hydrogen diffuses within the metal, creating a concentration gradient. Hydrogen atoms migrate from a region of high concentration on the metal surface to a lower concentration region within the metal’s interior. This description is based on Fick’s laws of diffusion.

Fick’s first law relates the diffusive flux, or, the rate at which a substance moves, to the concentration gradient Eq. (5):

5 $$J=-D\frac{\partial c}{\partial x}$$

In Eq. (6), $\partial c/\partial t$ represents the rate of change in concentration over time (Fick’s second law), where $D$ denotes the diffusion coefficient, and ${\partial }^{2}c/\partial {x}^{2}$ is the second derivative of the concentration with respect to distance.

6 $$\frac{\partial c}{\partial t}=D\frac{{\partial }^{2}c}{\partial {x}^{2}}$$

The permeation parameters can be calculated using Fick’s first and second laws with the following boundary conditions Eq. (7). Prior to the start of the experiment, the hydrogen concentration at both sides of the sample is zero. In a steady-state scenario, both the hydrogen flux and permeation current stabilize, reaching a plateau. At this point, the sub-surface hydrogen concentration on the charging side (where x = 0) also attains its maximum or achieves a steady-state value. Simultaneously, on the oxidation side (where x = L), all diffused hydrogen atoms surrender their electrons, transforming into hydrogen ions. This process generates an oxidation current, ensuring that the hydrogen concentration on the oxidation side remains zero. Therefore, by utilizing Fick’s first and second laws, the Eqs. (8)–(13) can be derived^11,80,81^.

7 $$\left\{\begin{array}{l}t=0,\,c\left(x,0\right)=0\\ t > 0,\,c\left(0,t\right)={C}_{0R}\\ t > 0,\,c\left(L,t\right)=0\end{array}\right.$$

The steady-state electrochemical permeation flux, ${J}_{{SS}}\,({mol}{\cdot }{m}^{-2}{s}^{-1})$:

8 $${J}_{{SS}}=\frac{{I}_{{SS}}/A}{F}=\frac{{D}_{{eff}}{C}_{0R}}{L}$$

Sub-surface concentration, ${C}_{0R}\,(mol\,\cdot \,{m}^{-3})$:

9 $${C}_{0R}=\frac{{J}_{{SS}}L}{{D}_{{eff}}}=\frac{{I}_{{SS}}L}{{FA}{D}_{{eff}}}$$

Effective diffusion coefficient, ${D}_{eff}\,({m}^{2}\,\cdot \,{s}^{-1})$:

Time lag method:

10 $${D}_{{eff},{tlag}}=\frac{{L}^{2}}{6{t}_{{lag}}}$$

Breakthrough time method:

11 $${D}_{{eff},{tb}}=\frac{{L}^{2}}{15.3{t}_{b}}$$

**Fig. 19:**
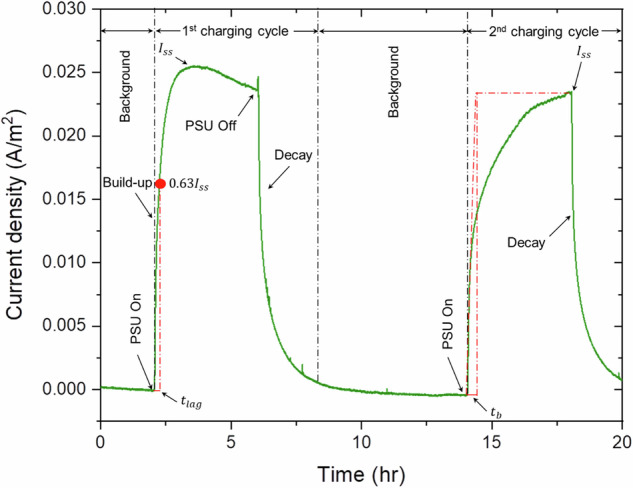
Representative hydrogen permeation current profiles for the first and second hydrogen charging cycles. The figure shows the evolution of permeation current over time, highlighting transient behavior and changes between cycles.

In these equations, the term ${I}_{{SS}}$ denotes the steady-state permeation current value, which is measured at the point when the permeation curve rises and stabilizes. $L$ signifies the thickness of the specimen, while $A$ stands for the contact area between the specimen and the electrolyte. $F$ is the symbol for Faraday’s constant ($96485\,{C\cdot }{{mol}}^{-1}$). The effective diffusion coefficient, ${D}_{{eff}}$, can be calculated in Eqs. (10) and (11). In Eqs. (10) and (11), by extrapolating the linear part of the permeation curve, we can obtain ${t}_{b}$, while ${t}_{{lag}}$ corresponds to the time taken to reach 63% of ${I}_{{SS}}$^79,80,82^. These parameters are illustrated in Fig. 19.

The process of determining the densities of reversible and irreversible traps begins with the first permeation curve. Based on Eqs. (10) and (11), the effective diffusion coefficient, ${D}_{{eff},1}$ can be derived. This allows for the determination of ${N}_{T,1}$, which represents the combined density of both trap types, as both participated in the initial charging process. For the second hydrogen charging, the permeation curve was used to calculate ${N}_{T,2}$. This value represents the density of only reversible hydrogen traps as the irreversible traps were filled during the first charging cycle. By subtracting ${N}_{T,2}$ from ${N}_{T,1}$, the density of the irreversible traps was identified. This method facilitates the separation of the densities of reversible and irreversible hydrogen traps^53,83^. In Eq. (12), $n$ is Avogadro’s number ($6.022\,\times \,{10}^{23\,}\,{{mol}}^{-1}$) and ${D}_{l}$ denotes the lattice diffusion coefficient. ${D}_{l}$ is often estimated as $1.28\times {10}^{-8}\,{m}^{2}\cdot {s}^{-1}$, which is the lattice diffusion coefficient of $\alpha -{Fe}$ ^43,46,82–84^.

The total density of hydrogen-trapping sites, ${N}_{T}\,({m}^{-3})$:

12 $${N}_{T}=\frac{n{C}_{0R}}{3}\left(\frac{{D}_{l}}{{D}_{{eff}}}-1\right)$$

The density of reversible hydrogen-trapping sites, ${N}_{{rev}}\,({m}^{-3})$:

13 $${N}_{T,2}={N}_{{rev}}$$

The density of irreversible hydrogen-trapping sites, ${N}_{{ir}}\,({m}^{-3})$:

14 $${N}_{{irr}}={N}_{T,1}-{N}_{{rev}}$$

### Electron backscatter diffraction (EBSD) analysis

The analysis of dislocations can provide valuable insights into growth defects in single crystals. This is particularly important from a hydrogen embrittlement perspective, as dislocations can serve as potential trap sites for hydrogen. Dislocations accumulate within a crystal during plastic deformation either to ensure the compatible deformation of different regions of the specimen or through random interactions with one another^85^. In both scenarios, there is a geometric relationship between the deformation and the number of dislocations required to accommodate it while minimizing internal stress^86^.

Each dislocation in the crystal lattice induces a slight shift in the orientation of atomic rows, though this change is typically too minor to detect. However, when multiple dislocations of the same sign accumulate, they cause a measurable change in lattice orientation or curvature. Dislocations of mixed signs are commonly known as statistically stored dislocations (SSDs). In contrast, dislocations of the same sign that contribute to the overall lattice curvature are termed geometrically necessary dislocations (GNDs). These GNDs can be measured using electron backscatter diffraction (EBSD) to analyze local orientations at specific points on a specimen’s planar surface^87,88^.

In this study, EBSD was used to determine the dislocation densities in pipeline steels. The EBSD module was operated with an Apreo 2S SEM (ThermoFisher) at an accelerating voltage of 25 kV, a current of 13 nA, and a working distance of 15–16 mm, with a step size of 0.203 µm between points. The specimens were positioned at a 70° tilt. To ensure comparability, the scanned area was kept consistent across all samples.

## Data Availability

All data supporting the findings of this study are included in the manuscript. No additional data were generated or analyzed beyond those presented.
